# Factors influencing uptake of protective behaviours by healthcare workers in England during the COVID-19 pandemic: A theory-based mixed-methods study

**DOI:** 10.1371/journal.pone.0299823

**Published:** 2024-05-09

**Authors:** Carly Meyer, Elise Crayton, Abigail Wright, Moira Spyer, Nina Vora, Catherine Houlihan, Naomi F. Walker, Eleni Nastouli, Susan Michie, Fabiana Lorencatto, on behalf of the SAFER investigators

**Affiliations:** 1 Centre for Behaviour Change, University College London, London, United Kingdom; 2 NIHR Policy Research Unit in Behavioural Science, University College London, London, United Kingdom; 3 Department of Infection, Immunity and Inflammation, UCL GOS Institute of Child Health, London, United Kingdom; 4 UCL Centre for Clinical Research in Infection and Sexual Health, Institute for Global Health, University College London, London, United Kingdom; 5 Department of Clinical Virology, University College London Hospitals NHS Trust, London, United Kingdom; 6 Department of Clinical Sciences, Liverpool School of Tropical Medicine, Liverpool, United Kingdom; 7 Tropical and Infectious Diseases Unit, Liverpool University Hospitals NHS Foundation Trust, Liverpool, United Kingdom; University of Haifa, ISRAEL

## Abstract

**Background:**

Hospital infection control policies protect patients and healthcare workers (HCWs) and limit the spread of pathogens, but adherence to COVID-19 guidance varies. We examined hospital HCWs’ enactment of social distancing and use of personal protective equipment (PPE) during the COVID-19 pandemic, factors influencing these behaviours, and acceptability and feasibility of strategies to increase social distancing.

**Methods:**

An online, cross-sectional survey (n = 86) and semi-structured interviews (n = 22) with HCWs in two English hospitals during the first wave of the COVID-19 pandemic (May-December 2020). The Capability, Opportunity, Motivation (COM-B) model of behaviour change underpinned survey and topic guide questions. Spearman Rho correlations examined associations between COM-B domains and behaviours. Interviews were analysed using inductive and deductive thematic analysis. Potential strategies to improve social distancing were selected using the Behaviour Change Wheel and discussed in a stakeholder workshop (n = 8 participants).

**Results:**

Social distancing enactment was low, with 85% of participants reporting very frequently or always being in close contact with others in communal areas. PPE use was high (88% very frequently or always using PPE in typical working day). Social distancing was associated with Physical Opportunity (e.g., size of physical space), Psychological Capability (e.g., clarity of guidance), and Social Opportunity (e.g., support from managers). Use of PPE was associated with Psychological Capability (e.g., training), Physical Opportunity (e.g., availability), Social Opportunity (e.g., impact on interactions with patients), and Reflective Motivation (e.g., beliefs that PPE is effective). Local champions and team competition were viewed as feasible strategies to improve social distancing.

**Conclusions:**

It is valuable to understand and compare the drivers of individual protective behaviours; when faced with the same level of perceived threat, PPE use was high whereas social distancing was rarely enacted. Identified influences represent targets for intervention strategies in response to future infectious disease outbreaks.

## Introduction

The COVID-19 pandemic placed unprecedented burden on health systems and communities globally. Healthcare workers (HCWs) on the frontline play a crucial role in managing the health impacts of COVID-19 but in doing so put themselves at increased risk of contracting COVID-19 [1–3] and experiencing psychological distress [4, 5]. To protect patients and HCWs and limit the spread of COVID-19 within hospital settings, national and local health authorities produced guidance on infection control for HCWs specific to COVID-19 [e.g.,^6^–^8^]. Many include recommendations about personal protective behaviours [9, 10], including donning and doffing personal protective equipment (PPE) such as masks, gowns, eye protection and visors; regular hand washing; social distancing (sometimes called physical distancing and defined as maintaining a 1–2 metre distance from other people); and disinfecting surfaces [6, 7, 11].

Evidence from past infectious disease outbreaks highlights the challenges of implementing such guidance on infection control measures within healthcare settings [12, 13]. In the UK, a national survey of 831 HCWs conducted in June 2020 identified low adherence to protective behaviours during the COVID-19 pandemic, with 80% adherence for PPE use, 67.8% for hand hygiene, and 74.7% for social distancing in communal areas [14]. In the US, adherence to PPE guidelines was found to vary depending on the clinical scenario, unexpectedly being highest for ‘patient contact when COVID-19 was not suspected’ and lowest when ‘carrying out aerosol generating procedures’ [15, 16]. In a survey of French primary care physicians, non-adherence to preventative behaviours (never or rarely wearing a mask and/or often or always hugging/shaking hands) was reported to be 7.2% [17]. To improve adherence to infection control guidelines, we need to understand what factors are influencing these behaviours, as a first step towards designing interventions likely to be effective in changing these behaviours in the event of future infectious disease outbreaks or pandemics [18, 19].

**Fig 1:**
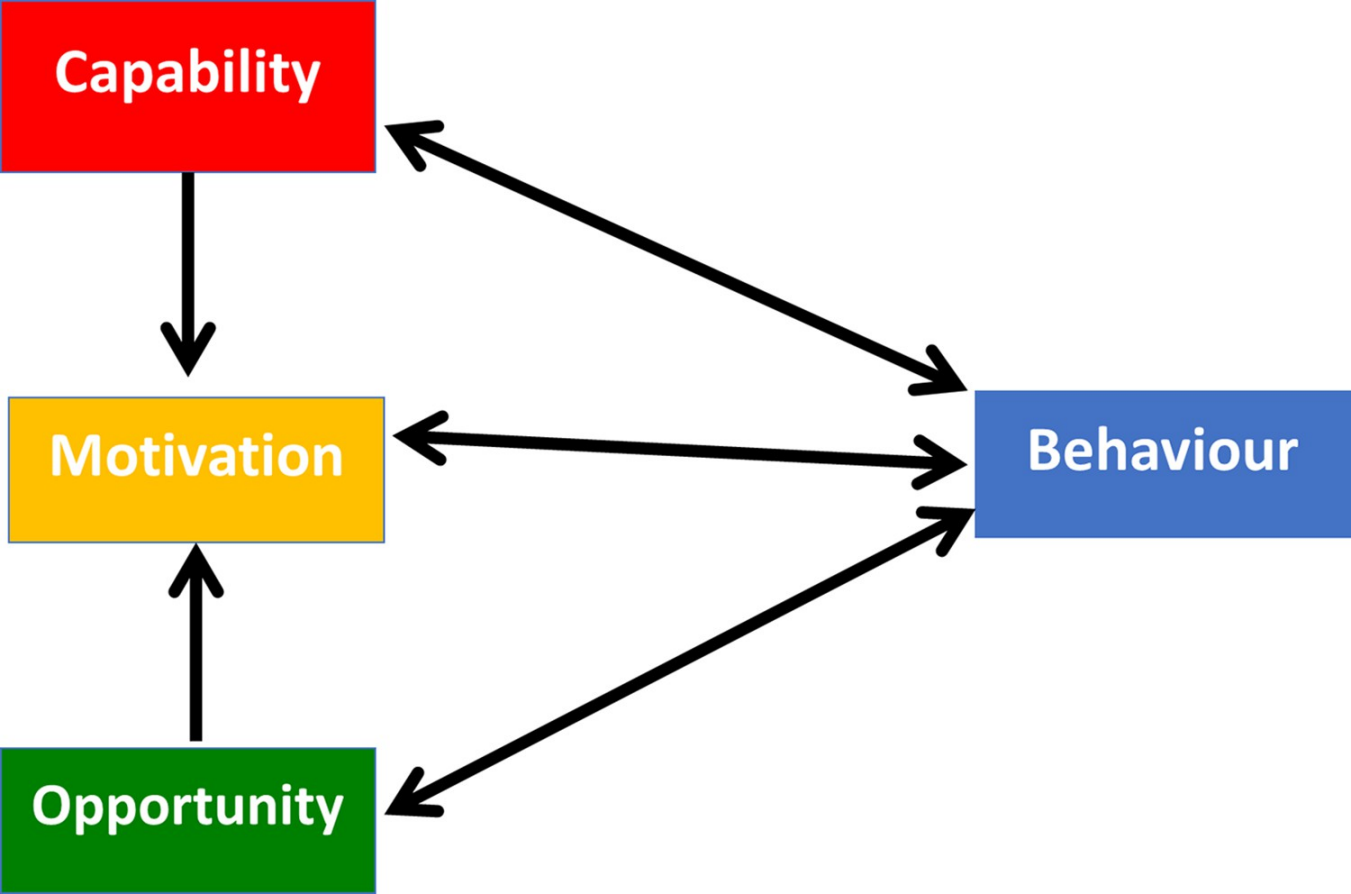
The COM-B model. Adapted from Michie, S., Atkins, L., & West, R. (2014). The behaviour change wheel: A guide to designing interventions. UK: Silverback Publishing.

In this study, we investigated behavioural influences using an integrative framework, the Behaviour Change Wheel (BCW) [18, 19]. At the core of the BCW is the Capability, Opportunity, Motivation model of Behaviour (COM-B), which specifies that for an individual to perform a Behaviour, they require physical and psychological capability (e.g., skills, knowledge), physical and social opportunity (e.g., time, physical space, social pressure), and reflective and automatic motivation (e.g., beliefs, emotions, habits) [18, 19] (Fig 1). For example, in the context of the COVID-19 pandemic, West et al. [10] described social distancing behaviour as requiring an understanding of why distancing is important (capability), a shift in social norms and physical layout (opportunity), and a ‘need’ to perform the behaviour (motivation).

There is a paucity of theory-informed research examining factors that drive HCWs’ use of protective behaviours. Among the general public in the UK, adherence to Government-enforced COVID-19 protective measures has been found to be associated with greater perceived Capability (e.g., being physically and psychologically able to follow government instructions), Opportunity (e.g., having the physical and social opportunity to follow government instructions), and Motivation (e.g., being motivated to follow government instructions) to perform the behaviour [20]. Similarly, among the general public in the USA, greater adherence to COVID-19 protective measures was associated with greater perceived threat, perceived control, and knowledge as conceptualised by the Health Belief Model [21]. Further, in a survey study during the early stages of the pandemic of the general public across 8 geographical locations (including parts of Asia, North and South America, and Europe), authors identified that prosociality, self-efficacy, and perceived susceptibility and severity of COVID-19 significantly affected adherence to protective behaviours [22]. To our knowledge, only the Smith et al. [14] HCW survey cited above has examined psychological influences on the use of protective behaviours among HCWs. Although, it should be noted that only 10 perent of their survey respondents had regular exposure to patients with COVID-19. PPE use and social distancing were both associated with adequate training, believing that there is a point in using protective measures if they have extensive contact with COVID-19 patients, feeling safe at work, and more favourable attitudes towards protective measures among colleagues [14]. PPE use increased with clear guidance on what PPE to use and when, appropriate PPE supplies, perceiving that PPE allowed one to do the job properly, and feeling less anger about how PPE was distributed. Social distancing in communal areas was associated with the receipt of credible information from the National Health Service (NHS) about PPE, the use of clear environmental markings, supportive workplace design, and perceived ease of social distancing at work. Handwashing was influenced by the accessibility of handwashing facilities and perceived risk of transmitting COVID-19 to friends and family [14]. These factors are similar to those identified during past infectious disease outbreaks (e.g., H1N1, SARS, MERS) [12, 13].

Our study is the first to use qualitative methodology and an overarching theoretical framework to address the question of psychological, social, and environmental influences on the use of protective behaviours in HCWs. We focused on two key behaviours: social distancing in non-clinical communal areas (including meeting rooms, canteens, social spaces/staff rooms and offices) and the use of PPE. The use of PPE was seen as instrumental in preventing COVID-19 transmission during clinical care; however, it was recommended that staff remain socially distanced in communal areas when PPE use was not mandated or was impractical (meal times) to limit staff-to-staff transmission. The use of qualitative methodology combined with a quantitative survey of two specific hospital sites in England allows granular exploration of influences that could be targeted by interventions. This study addressed three research questions:

1. To what extent did hospital healthcare staff in two large UK teaching hospitals enact personal protective behaviours (social distancing in communal areas and use of PPE) during the COVID-19 pandemic?
2. What capability, opportunity, and motivational factors influenced social distancing in communal areas and the use of PPE?
3. What strategies are acceptable and feasible to increase adherence to recommendations for social distancing in communal areas?

## Methods

This study was part of a larger study called SARS-CoV-2 Acquisition in Frontline Healthcare Workers—Evaluation to inform Response (SAFER), a prospective cohort study of 300 frontline healthcare works across two acute NHS city-based hospital trusts [3]. Ethical approval was granted by the South Central—Berkshire Research Ethics Committee (Ref 20/SC/0147).

### Study design

A mixed-methods study incorporating an online, cross-sectional survey and semi-structured interviews (Phase 1), and a stakeholder intervention design workshop (Phase 2). Data were collected during the first and second wave of the COVID-19 pandemic (May-December, 2020), prior to COVID-19 vaccine approval and roll-out in the UK.

Phase 1

#### Cross-sectional survey. *Participants and sampling*

Participants were recruited from two UK urban acute hospital trusts participating in the SAFER study. HCWs were eligible to participate in SAFER if they had worked in at least one of the following clinical areas during the study period: accident and emergency (A&E), haematology, infectious diseases, acute medicine, intensive care unit (ICU), COVID-19 cohort wards, and other general medical wards. Participants included clinical and non-clinical staff (e.g., doctors, nurses, healthcare assistants, physiotherapists, porters, housekeeping staff, catering staff, and administrative staff). Enrolment in the Behavioural study was by invitation extended to all SAFER participants.

#### Procedure

Participants were informed of the study via SMS messages and/or email with up to two reminders, and by QR codes in waiting rooms for participants in SAFER [3]. Upon clicking the survey link, participants were given study information and a consent form. The survey was hosted on REDCap [23]. No incentives for completing the survey were offered.

The survey included items to assess participant demographics (e.g., gender, ethnicity); healthcare worker role; COVID-19 exposure and perceived risk; extent of social distancing, e.g., “*In one day*, *how often do you find yourself in close proximity (i*.*e*., *less than 6ft/2m) to other staff members when in communal / non-clinical areas*?” (Always, Very Frequently, Sometimes, Occasionally, Rarely, Never); use of PPE, e.g., “*In a typical working day*, *how often do you use PPE*?” (Always, Very Frequently, Occasionally, Rarely, Never); and barriers and enablers to enacting these behaviours whilst working in hospital settings (see S1 Table for overview of behavioural questions in the survey).

The latter included items developed specifically for this study structured around the domains of the COM-B model [18, 19], with at least one item per domain in the form of belief statements to which participants rated their agreement on a Likert-type scale from 1 (Strongly Disagree) to 5 (Strongly agree). For instance, the statement ‘*Furniture in communal areas is too close together*’ aimed to assess the role of Physical Opportunity in influencing social distancing behaviour (see Table 1). The COM-B scale for social distancing included 27 items, with statements exploring influences related to Psychological Capability (n = 5), Social (n = 6) and Physical (n = 5) Opportunity, and Reflective (n = 5) and Automatic (n = 5) Motivation. Following the removal of one item (‘My colleagues would disapprove if I didn’t keep 2m/6ft away from them’), each subscale had good internal consistency indicating all items within a single scale were measuring the same construct and thus subscale scores can be computed (Cronbach’s alpha = 0.67–0.77; see Table 3). The COM-B scale for PPE use included 24 items, with statements exploring influences related to Physical (n = 2) and Psychological (n = 4) Capability, Social (n = 5) and Physical (n = 6) Opportunity, and Reflective (n = 5) and Automatic (n = 2) Motivation. Good internal consistency was recorded for four of the six subscales (Cronbach’s alpha = 0.62–0.77); however, low internal consistency was recorded for Reflective Motivation (Cronbach’s alpha = 0.50) and Automatic Motivation (Cronbach’s alpha = 0.27), suggesting that these subscales are more heterogenous and possibly measuring different constructs, and therefore subscale scores can not be computed (see Table 5).

**Table 1:** Overview of behavioural questions in survey and qualitative interviews.

COM-B Domain	Social Distancing		Wearing Personal Protective Equipment (PPE)	
	Survey	Interview	Survey	Interview
Capability–Physical	n/a	n/a	I have had insufficient training on PPE use	How easy or difficult is it to put on/remove PPE safely?
Capability–Psychological	The guidance on distancing in communal staff areas is unclear	How easy or difficult is it to judge if you are 2m/6ft apart from colleagues?	The guidance on when and how to use PPE is unclear	In the context of Covid-19, how clear are you on when and how you should use PPE?
Opportunity–Social	Keeping physically apart from my colleagues interferes with team morale and culture	How does distancing in communal areas impact on your relationship with colleagues?	My colleagues are not using PPE	Do your colleagues tend to use PPE as indicated? To what extent is wearing PPE the norm among your colleagues?
Opportunity–Physical	Furniture in communal areas is too close together	How much does the layout of communal areas influence whether or not it is possible to socially distance?	There are limited supplies of PPE	To what extent are PPE supplies readily available in this hospital?
Motivation–Reflective	There is less risk of COVID-19 in non-clinical, communal areas	How important do you think it is for you and your colleagues to maintain social distancing in communal areas (when it is physically possible)?	Wearing PPE interferes with my ability to deliver care	How effective do you think using PPE is at reducing risk of Covid-19?
Motivation–Automatic	I am not in a habit of keeping distance from colleagues	Have you developed any habits or routines to try and maintain social distancing from colleagues in communal areas?	Wearing PPE is painful or uncomfortable	How does using PPE make you feel?

#### Data analysis

Data were stored on REDCap and exported into STATA (Version 16) for analysis. For multiple choice and dichotomous yes/no responses, the number of respondents (n) and percentage of respondents (%) were calculated per response option. For Likert scales, mean item scores and standard deviations were calculated. Incomplete survey responses were omitted from analysis if only section 1 (healthcare worker role) was answered.

Two ordinal logistic regression models were planned at the study outset to explore relationships between the use of protective behaviours and COM-B influences on behaviour. Due to small sample sizes and highly skewed outcome measures, multivariate regression modelling could not proceed. Instead, a series of Spearman Rho correlations were computed to explore associations between (1) social distancing behaviour and individual and subscale COM-B items; and (2) use of PPE on a typical working day and individual and subscale COM-B items. Small samples sizes also prevented the capacity to explore differences between sites.

#### Qualitative interviews. *Participants and sampling*

A subsample of HCWs who completed the online survey was purposively sampled to represent a range of different clinical and non-clinical healthcare roles across the various clinical areas. We aimed to recruit a minimum of 13 participants as per guidance for theory-based interview studies [24] and to continue data collection until no new emerging themes arose from the data (thematic saturation).

#### Procedure

Individuals who completed the online survey could indicate their interest to participate in follow up individual, semi-structured interviews by entering their contact details. Potential participants were selected according to the purposive sampling strategy and were invited to take part in a telephone or video interview. Participants provided consent in writing and verbally before the interview commenced. Interviews were audio recorded and transcribed verbatim. Interviews were conducted by two researchers trained in qualitative methods and application of behavioural science frameworks (CM (PhD Speech Pathologist) and EC (PhD Health Psychologist)) and lasted an average of 56 minutes (range 40–77 minutes).

#### Topic guides

The semi-structured interview topic guide (see S1 Fig) followed a similar format to the survey but included open-ended questions to explore behaviour and behavioural influences in more detail (see Table 1). The topic guide started by asking participants to provide some context about their role and typical working day followed by COM-B based questions to explore factors influencing the use of PPE and social distancing in non-clinical, communal areas. Topic guides were pilot tested by both interviewiers with one person each and refined before recruitment began.

#### Data analysis

Data were analysed using a rapid analysis approach that involved both deductive (guided by framework analysis methods [25]) and inductive thematic analysis. Verbatim transcripts were summarised by first applying a structured template (based on the interview guide sections and the COM-B model) and subsequently generating theme labels inductively within each section of the template. Prior to applying the template, it was piloted by three researchers (CM, EC, AW) to assess whether the template would help capture data that aligned with the COM-B model and research questions. All transcripts were analysed by one researcher (AW, MSc Health Psychologist) and 10% were double coded by two other members of the research team (CM and EC). Regular meetings were held to refine the analysis and resolve any disputes. Analysis was conducted on NVIVO software.

### Phase 2: Stakeholder workshop

Phase 2 focused on social distancing, which was identified as the target, priority behaviour needing change because it was found to be enacted infrequently in the survey and staff-to-staff transmission was identified as a key source of COVID-19 infection in one of the participating hospitals. Suggestions for strategies to address the barriers and enablers to social distancing that were identified in the Phase 1 survey and interviews were generated by consulting matrices [18] which pair the COM-B model with two frameworks of behaviour change intervention strategies: the BCW—which specifies nine broad intervention types (e.g. education, persuasion, environmental restructuring) [18, 19], and the Behaviour Change Techniques (BCTs) Taxonomy v1 [26]–which specifies 93 more granular BCTs (e.g. information about health consequences). These matrices suggest which intervention types and BCTs are more likely to be relevant and effective in addressing barriers and enablers within the different COM-B domains. Furthermore, the participating hospital provided the materials and descriptions of existing interventions they had implemented to try and improve social distancing amongst hospital staff during the COVID-19 pandemic. We conducted a content analysis by coding these materials into component BCTs using BCT Taxonomy v1 [26]. We assessed the gaps between BCTs delivered in existing interventions and those identified as potentially relevant and effective in the aforementioned mapping exercise, to generate recommendations for additional behaviour change strategies that could be implemented to address barriers/enablers and improve social distancing.

These recommendations were subsequently presented in an online stakeholder consultation meeting hosted on MS Teams and attended by a convenience sample of senior HCWs from a range of clinical (e.g., virologists) and non-clinical (e.g., administrators) roles at the participating hospital. Stakeholders discussed the feasibility of implementing each of the proposed intervention strategies using the APEASE criteria (Acceptability, Practicability, Effectiveness, Affordability, Safety/Side effects, and Equity) [18]. We also asked for suggested refinements to the intervention packages presented and suggestions for additional intervention strategies that could be considered. The meeting was audio-recorded. The meeting transcript and meeting chat were analysed by first deductively coding responses to domains of APEASE, then inductively generating themes within each domain.

## Results

### Phase 1: Understanding influences on behaviour

#### Participants

Eighty-six of the 300 participating HCWs in the wider SAFER study completed the online survey between May and August 2020, representing a response rate of 29%. Participant demographics are provided in Table 2. Most participants were clinical staff (41.9% nurses and 33.7% doctors). Each of the seven clinical areas targeted were represented in the data, with most participants working in A&E (33%), the ICU (24%), the Acute Medical Unit (21%), and Haematology (21%). Sixteen percent of the total sample reported receiving a confirmed diagnosis of COVID-19 before completing the survey; however, 86% reported that a team member had received a positive diagnosis. Approximately 63% of participants reported that their likelihood of coming into contact with a patient with COVID-19 was very likely or definite. Moreover, a similar proportion of participants reported that caring for COVID-19 patients was a substantial or main part of their role. The majority (71%) of participants reported perceptions that they were at moderate risk or greater of contracting Covid-19. See S3 Table for further information.

**Table 2:** Participant characteristics of survey respondents (N = 86).

Variable			
Age—M (SD)		38.9	(22.2)
Gender–N (%)			
	Female	59	(71.9%)
	Male	24	(28.9%)
Ethnicity–N (%)			
	White	63	(75.9%)
	Asian or Asian British	9	(10.8%)
	Black or Black British	4	(4.8%)
	Mixed	4	(4.8%)
	Ashkenazi Jewish	1	(1.2%)
	Mauritian	1	(1.2%)
	Other	1	(1.2%)
Role–N (%)			
	Doctor (all)	29	(33.7%)
	*- Consultant*	8	(9.3%)
	*- Postgraduate doctor in training*	15	(17.4%)
	*- Other*	6	(7.0%)
	Nurse	36	(41.9%)
	Advanced Clinical Practitioner	3	(3.5%)
	Healthcare Assistant	7	(8.1%)
	Physiotherapist	2	(2.3%)
	Therapy Assistant	1	(1.2%)
	Student Nurse	1	(1.2%)
	Administrator	3	(3.5%)
	Porter	1	(1.2%)
	Housekeeper	2	(2.3%)
	Missing	1	(1.2%)
Time in role–N (%)			
	Less than 1 month	2	(2.3%)
	1 to 12 months	28	(32.6%)
	1 to 4 years	24	(27.9%)
	5 to 9 years	10	(11.6%)
	10 years +	22	(25.6%)
Time at hospital–N (%)			
	1 to 12 months	24	(27.9%)
	1 to 4 years	24	(27.9%)
	5 to 9 years	15	(17.4%)
	10 years +	23	(26.7%)
Clinical area–N (%)			
	Accident & Emergency	28	(32.6%)
	Haematology	18	(20.9%)
	Infectious Diseases	13	(15.1%)
	Acute Medical Unit	18	(20.9%)
	Intensive Care Unit	21	(24.4%)
	COVID-19 Cohort Ward	11	(12.8%)
	Other	13	(15.1%)
	N/A	3	(3.5%)

A subsample of 48 participants expressed interest and were contacted about interviews. Twenty-six participants either did not respond to contact about the interview (n = 22) or did not attend the scheduled interview (n = 4). Twenty-two HCWs (mean age = 39.9, SD = 10.5; 64% female) subsequently participated in interviews which were conducted between June and August 2020. Most (73%) interviewees were White, with others identifying as Asian or Asian British (n = 2), Black or Black British (n = 2), mixed (n = 1), or Ashkenazi Jewish (n = 1). Participants represented doctors (n = 8), nurses (n = 6), advanced clinical practioners (n = 3), healthcare assistants (n = 2), administrators (n = 2), and porters (n = 1). Participants worked across a number of wards and specialisms including: haematology (n = 2), surgery (n = 1), ICU (n = 3), A&E/emergency ambulatory clinics (n = 6), Infectious disease (n = 2), acute medicine (n = 3), and multiple wards (n = 5).

The findings from the interviews and surveys are concurrently discussed below. Identifiers are used alongside quotes; however, in order to protect participant anonymity, we have removed ward name in some cases.

#### Using social distancing

Overall, maintenance of social distancing in communal areas was low, with 84.9% of survey respondents reporting that they came into close contact (<2m) with colleagues in communal areas ‘very frequently’ or ‘always’ (S3 Table). Social distancing was reported by many to be difficult during handovers (80%), break/rest times (78%), mealtimes (67%), and meetings (59%). Social distancing was perceived to be difficult in all communal areas, except for toilets, canteens/cafes/restaurants, and outside the building (S2 Table). When social distancing was not possible, participants most commonly performed handwashing with soap (83%) or alcohol rub (80%), avoided touching their face (79%), disinfected objects and surfaces (65%), or used PPE (33%) (S3 Table).

A summary of COM-B influences on social distancing behaviour are presented in Tables 3 and 4, with the former displaying the results from the survey and the latter presenting the subthemes derived from the qualitative interviews. Additional supporting quotes can be found in S5 Table.

**Table 3:** Agreement with COM-B items related to social distancing in communal areas and Spearman rho (rs) correlations with coming into close contact with others.

Scale/Item	Mean	SD	N	r_s_
**Capability-Psychological**	**2.83**	**0.750**	**82**	**0.234** *
I often forget to keep 2m/6ft apart from colleagues	3.01	1.167	84	0.095
I have not been told to stay 2m/6ft apart from colleagues when in communal areas	2.39	1.242	84	0.223*
The guidance on distancing in communal staff areas is unclear	2.87	1.220	84	0.099
I am too busy or in a hurry to think about distancing from colleagues	3.06	1.034	84	0.244*
It is hard to judge whether I am maintaining a 2m/6ft distance	2.81	1.012	82	0.101
*Cronbach’s alpha*	**0.67**			
**Opportunity- Social**	**2.92**	**0.735**	**83**	**0.197**
There is lack of support or encouragement from managers to maintain a 2m/6ft distance	2.70	1.159	84	0.255*
Others around me are not maintaining a 2m/6ft distance	3.49	0.942	83	0.145
Keeping physically apart from my colleagues interferes with team morale and culture	3.17	1.085	84	0.045
There is lack of support or encouragement from peers to maintain a 2m/6ft distance	3.10	1.025	84	0.185
I am worried about how my colleagues would react if I tried to keep away from them	2.17	1.046	83	0.141
Distancing in communal areas is not normal or expected	2.87	1.197	83	0.051
*Cronbach’s alpha*	**0.77**			
**Opportunity-Physical**	**3.72**	**0.761**	**84**	**0.205**
Distancing is impractical or difficult to do	3.68	1.077	84	0.156
There is not enough space in communal areas to maintain a 2m/ft distance	4.01	1.114	84	0.233*
Furniture in communal areas is too close together	3.66	1.036	84	0.136
Communal areas are overcrowded	3.62	1.040	84	0.187
We have nowhere else to go with more space for breaks and meetings	3.64	1.258	84	0.172
*Cronbach’s alpha*	**0.72**			
**Motivation- Reflective**	**2.34**	**0.667**	**82**	**0.062**
I personally do not feel at risk of catching COVID-19	2.20	0.954	84	0.005
There is less risk of COVID-19 in non-clinical, communal areas	2.00	0.944	84	-0.071
Distancing among staff is not a priority	2.74	1.004	82	0.112
I don’t see my colleagues as a risk	2.34	1.016	83	0.018
Keeping 2m/6ft apart from my colleagues won’t help reduce COVID-19 risk	2.43	1.123	84	0.061
*Cronbach’s alpha*	**0.67**			
**Motivation–Automatic**	**3.09**	**0.724**	**83**	**0.070**
I am not worried about catching COVID-19	2.46	1.207	84	-0.006
I am not in a habit of keeping distance from colleagues	3.20	1.117	84	0.074
When I am in communal areas, I just want to relax	3.55	1.057	84	0.175
I enjoy being close to my colleagues	3.16	0.943	83	0.069
It is awkward to keep apart from my colleagues	3.11	1.172	84	0.061
*Cronbach’s alpha*	**0.67**			

*Note*: Item scoring: (1 = strongly disagree; 5 = strongly agree)

*p = <0.05

**Table 4:** Subthemes related to use of social distancing in communal areas.

COM-B Domain and Subtheme		Type of Influence	Frequency
**Psychological Capability**			
	Clarity of guidance on social distancing	Mixed	15
	You have to keep social distancing in mind all the time	Barrier	15
	Varying ability to judge 2m distance	Mixed	15
	Increased awareness of social distancing	Enabler	6
	Social distancing guidance introduced too late	Barrier	4
	Information overload	Barrier	2
**Social Opportunity**			
	Peer pressure	Mixed	7
	Peer acceptance of social distancing	Enabler	4
	Less likely to distance from colleagues they are more familiar with	Barrier	4
	Personal cultural norms are discordant with social distancing	Barrier	4
	Social distancing impacts workplace culture	Barrier	3
	Role modelling	Mixed	3
	Managers support social distancing	Enabler	2
	Feedback from peers	Enabler	1
**Physical Opportunity**			
	Size of physical space	Barrier	17
	Layout of furniture and office equipment	Mixed	17
	Limiting staff numbers	Enabler	12
	Access to larger physical spaces	Enabler	10
	Social distancing guidance was impractical	Barrier	10
	Inconsistent implementation of distancing rules	Barrier	9
	Floor markings prompt social distancing	Enabler	8
	Virtual working	Enabler	7
	Lack of time	Barrier	3
**Reflective Motivation**			
	Impact of social distancing on interactions and relationships with colleagues	Mixed	16
	Belief that poor adherence to social distancing can lead to COVID-19 outbreaks	Enabler	11
	Social distancing less effective than other protective behaviours	Barrier	7
	My colleagues aren’t a risk to me	Barrier	5
	Contradictory to socially distance in communal areas after not distancing in clinical areas	Barrier	4
	Too late for social distancing to make a difference	Barrier	4
	Social distancing is not a priority over job responsibilities	Barrier	1
	Impact on day-to-day workplace activities	Barrier	5
**Automatic Motivation**			
	Forming habits around social distancing	Mixed	6
	Seeking emotional support through human contact	Barrier	1
	Feeling overwhelmed	Barrier	1

*Psychological capability*. Overall mean scores on COM-B survey items pertaining to psychological capability were relatively neutral. Only two items were positively correlated with not maintaining social distancing: ‘I am too busy or in a hurry to think about distancing from colleagues’ (M = 3.06, SD = 1.03; R_s_ = 0.244, *p* = 0.027) and ‘I have not been told to stay 2m/6ft apart from colleagues when in communal areas’ (M = 2.39, SD = 1.24; R_s_ = 0.223, *p* = 0.044) (Table 3).

In interviews, some participants reported that there was clear, frequent guidance on social distancing that was helpful; however, others disagreed, commenting that the guidance did not provide advice on how to socially distance, where it should be performed, and how it should be used in combination with PPE. Participants reported it was difficult remembering to social distance, commenting “*you have to keep that in mind all the time*” (Healthcare Assistant, Acute Medicine, site 2). Participants reported forgetting to social distance, for example, when they were with familiar colleagues, when new guidance was introduced, and when there were no reminders to social distance. At times this was linked to the belief that social distancing is not important (reflective motivation): *"people will forget but I think that just means that they don’t intrinsically think that it’s something they think they should be doing"* (Doctor, Infectious Diseases, site 1). Furthermore, participants reported varying abilities to judge a 2-metre distance. Some reported it was easy, but others found it difficult, commenting “*two metres it’s farther than you’d think*” (Doctor, Acute Medicine, site 1).

*Social opportunity*. Only one item was positively correlated with not maintaining social distancing: ‘*There is lack of support or encouragement from managers to maintain a 2m/6ft distance*’ (M = 2.70, SD = 1.16; R_s_ = 0.255, *p* = 0.020) (see Table 3).

This is echoed in the interviews, where some participants reported that senior staff encouraged others to pay attention to social distancing and modelled social distancing behaviour; however, it was noted that social distancing was not often enforced, resulting in a lack of peer pressure: "*They mention that we should be doing it*, *but they … seem to turn a blind eye when it’s actually not happening*." (Nurse, A&E, site 1). For some participants, social distancing did not align with their cultural norms, with some members of staff from similar cultural background regularly sharing meals in communal areas. Likewise, social distancing was more challenging for HCWs from cultural backgrounds where physical contact is a defining feature: *“I’m Mediterranean*, *so I’m used to*, *to*, *to whoever*, *a certain type of physical contact with people around me”* (HCW, site 1). Peer acceptance of social distancing was considered an enabler: "*I think everyone is aware of it and if you said*, *oh*, *we need to be social distancing nobody would ever question it or disapprove of it*.” (Doctor, Acute Medicine, site 1).

*Physical opportunity*. Overall, the highest levels of agreement were recorded for the physical opportunity COM-B items (see Table 3). Participants agreed there was not enough space in communal areas to maintain a 2m/6ft distance, distancing was impractical or difficult to do, furniture in communal areas was too close together, there was nowhere else to go with more space for breaks and meetings, and communal areas were overcrowded. One item was positively correlated with not maintaining social distancing: ‘*There is not enough space in communal areas to maintain a 2m/ft distance*’ (M = 4.01, SD = 1.11; R_s_ = 0.233, *p* = 0.033).

In interviews, participants emphasised that the size of available space in the hospital was the main barrier to social distancing: “*it would very frequently be the case where there were more of us in there… to the point where you weren’t able to distance*” (Doctor, Haematology, site 2). Some participants noted that efforts were made to increase access to larger physical spaces and this helped facilitate social distancing: *"We’ve tripled our staff rest area*, *mainly because we have to keep two metres*, *people can’t wear masks while they’re eating"* (Manager, site 1). The layout of furniture and office equipment also hindered social distancing in the Hospital: *“Then there’s maybe two walls*, *two tables*, *maybe 1 x 1 metre so just… there’s not really any way more than four people could eat at those being socially distanced"* (Doctor, Acute Medicine, site 1). Re-arranging furniture and limiting the number of staff members permitted in communal areas was considered helpful. Although, participants commented that some guidance was impractical: " *there were signs about … only four people in a lift…*.. *Well*, *what are people supposed to do*?" (Nurse, ICU, site 1).

*Reflective motivation*. Participants most strongly disagreed with statements that fell within the domain Reflective Motivation; most participants disagreed with the statements that there was less risk of COVID-19 in non-clinical, communal areas, they were not at risk of catching Covid-19, their colleagues were not a risk, and social distancing from colleagues would not help reduce risk of contracting COVID-19 (see Table 3). No reflective motivation items were significantly correlated with social distancing behaviour.

In the interviews, many participants discussed the negative impact of social distancing on relationships with colleagues: “*it’s very important as a team*, *like*, *you*, *[laughs] you eat together*, *sit together*, *talk together*. *And if you’re splitting that up*, *or trying to make people go into different areas and different rooms*, *you’re*, *you’re breaking up the team*” (Doctor, Surgery, site 2). Some participants believed in the efficacy of social distancing as a protective behaviour, believing that social distancing can break the chain of hospital transmission and that poor adherence to social distancing can lead to COVID-19 outbreaks within the hospital: *"… why is it that now that’s when the staff are catching it and not the patient*? *So*, *we know that it’s probably something to do with staff getting too close to staff*. *That’s why it’s very important now to social distance because we’re trying to break that chain"* (Nurse, ICU, site 1). Other participants, however, indicated that social distancing may not be as effective as other protective behaviours and some participants viewing their colleagues as low risk because they work closely together clinically: “*we’re one bubble now*, *we’re one social bubble*.*”* (Manager, site 1).

*Automatic motivation*. Participants agreed that when they were in communal areas they just wanted to relax and disagreed with the statement that they were not worried about catching COVID-19 (see Table 3). No automatic motivation items were significantly correlated with social distancing behaviour.

In the interviews, participants reflected on how social distancing requires the breaking of existing habits: “*if you’ve had 20 or 30 or 40 years of*, *of being a bit tactile or yeah just not thinking about this stuff*, *it’s not easy to*, *to change that in one foul swoop*” (Nurse, ICU, site 1). Others reported that social distancing became a habit quickly, despite feeling awkward initially. Two participants noted that social distancing interfered with the need for emotional support through human contact when working through a pandemic: *“…we were all sleeping together on the couch and sofa… we are working harder*, *and… to relieve the stress… we are trying to stay closer somehow*.*”* (Doctor, site 1). One participant described feeling overwhelmed by the number of changes they needed to make, expressing that monitoring staff’s use of social distancing “*felt like one thing too many*” (Nurse, ICU, site 1).

#### Wearing PPE

PPE use was very high, with 88% of participants reporting that they ‘always’ or ‘very frequently’ use PPE in a typical working day (S4 Table). Three-quarters of participants reported wearing PPE during any patient contact; however, PPE was used less frequently when not working directly with patients (S3 Table), with 34% of participants using PPE when walking around the hospital and 17% when working at a desk. Less than 20% of participants reported wearing PPE ‘always’ or ‘very frequently’ in communal areas. The most frequently used PPE in any given task was face masks (88%), plastic aprons (85%), and gloves (84%) (S4 Table).

A summary of COM-B influences on using PPE are presented in Tables 5 and 6, with the former displaying the survey results and the latter presenting the subthemes derived from the qualitative interviews. Additional supporting quotes can be found in S6 Table.

**Table 5:** Agreement with COM-B items related to use of personal protective equipment (PPE) and Spearman rho (rs) correlations with using PPE.

Scale/item	Mean	SD	N	r_s_
**Capability–Physical**	**2.01**	**0.972**	**78**	**0.006**
I have had insufficient training on PPE use	2.05	1.031	78	0.029
I am not confident that I can put on and remove PPE safely	1.96	1.243	78	-0.063
*Cronbach’s alpha*	**0.62**			
**Capability–Psychological**	**2.43**	**0.817**	**77**	**0.000**
The guidance on when and how to use PPE is unclear	2.30	1.170	79	0.085
There has not been clear communication and information sharing within the hospital	2.26	1.081	77	0.003
Guidance around PPE use has been inconsistent	3.21	1.231	78	0.022
I sometimes forget to use PPE	1.99	1.145	78	-0.172
*Cronbach’s alpha*	**0.66**			
**Opportunity–Social**	**2.07**	**0.779**	**76**	**-0.022**
My colleagues are not using PPE	1.78	0.907	78	-0.266*
I lack support from managers	1.69	1.971	78	-0.064
I lack support from peers	1.64	0.842	77	-0.082
Colleagues in other roles need PPE more than I do	2.36	1.307	77	0.108
Using PPE makes patients feel isolated, afraid and/or stigmatised	2.82	1.266	78	-0.098
*Cronbach’s alpha*	**0.77**			
**Opportunity–Physical**	**2.42**	**0.715**	**77**	**-0.073**
The guidance and recommendations around PPE use are impractical or difficult to implement	2.58	1.038	78	0.041
My PPE does not fit properly	2.23	1.224	77	0.147
Using PPE Increases my workload (e.g. donning/doffing PPE, additional cleaning)	3.01	1.324	78	0.000
There are limited supplies of PPE	2.76	1.379	78	-0.123
PPE is not available in a convenient location	2.04	1.044	77	-0.174
I don’t have enough time to put on/remove PPE	1.86	0.922	78	0.008
*Cronbach’s alpha*	**0.67**			
**Motivation–Reflective**	**-**	**-**	**-**	**-**
The available PPE is not of an appropriate standard to ensure safety for staff and patients	2.64	1.347	77	0.026
Wearing PPE interferes with my ability to deliver care	2.51	1.182	78	-0.120
Using PPE is not a priority compared to other things I have to do when caring for patients	1.71	0.886	77	-0.148
PPE is not necessary in my role	1.54	0.817	78	-0.077
I am not sufficiently at risk or exposed to COVID-19	1.68	0.890	78	-0.038
*Cronbach’s alpha*	**0.50**			
**Motivation–Automatic**	**-**	**-**	**-**	**-**
Wearing PPE is painful or uncomfortable	3.06	1.283	78	0.082
I am not in the habit of using PPE	1.87	0.998	78	-0.146
*Cronbach’s alpha*	**0.27**			

*Note*: Item scoring: (1 = strongly disagree; 5 = strongly agree)

*p = <0.05

**Table 6:** Subthemes related to use of personal protective equipment (PPE).

COM-B Domain and Subtheme		Type of Influence	Frequency
**Psychological Capability**			
	Clarity and consistency of PPE guidance within and across Trust	Mixed	17
	Frequently changing PPE guidance	Mixed	10
	Signage informed staff about what PPE to be used	Enabler	10
	Forgetting to wear recommended PPE	Barrier	10
	Actively seeking evidence-based information on PPE use	Enabler	2
**Physical Capability**			
	Training in PPE use	Mixed	16
	Having the necessary skills to don and doff PPE	Enabler	14
	Training less relevant due to guidance changes	Neutral	4
**Social Opportunity**			
	Impact of PPE on interactions with patients	Mixed	19
	Peer support	Enabler	8
	Peer pressure	Mixed	5
	Role modelling	Enabler	5
**Physical Opportunity**			
	PPE supplies	Mixed	18
	PPE accessibility	Mixed	12
	Fit of PPE	Mixed	9
	Mandating use of face masks in non-clinical areas	Enabler	6
	Time to don and doff PPE as recommended	Mixed	5
	Quality of PPE	Neutral	4
**Reflective Motivation**			
	PPE is effective and makes you feel safe	Enabler	22
	PPE impact on clinical care	Mixed	12
	PPE negatively impacts interactions with colleagues	Neutral	9
	Use of PPE is generating excessive environmental waste	Neutral	5
	Lack of faith in recommendations for PPE use	Barrier	3
	People feel a false sense of protection	Barrier	1
	Wearing PPE is unnecessary for non-clinical tasks	Barrier	1
**Automatic Motivation**			
	PPE is uncomfortable to wear	Neutral	17
	Wearing PPE has become habit	Enabler	6
	Wearing PPE can make you stressed and anxious	Neutral	3
	Wearing PPE can leave you feeling thirsty or in need of a comfort break	Neutral	3
	Bored of wearing PPE	Neutral	2

*Psychological and physical capability*. Participants’ survey responses indicated they were confident they could put on and remove PPE safely and received sufficient training on PPE use. Likewise, participants disagreed with the statement that they sometimes forgot to use PPE and indicated there had been clear information sharing in the hospital and clear guidance on when and how to use PPE, although there was some agreement with the statement that guidance on PPE use was inconsistent. No significant correlations with PPE use were identified (see Table 5).

In interviews, some participants reported that specific guidance on PPE use that is easily accessible and frequently updated is an enabler to using PPE; however, frequent changes to guidance made adherence challenging and impacted staff confidence that guidance was evidence-based. Other challenges associated with PPE guidance included the high volume of information and inconsistency across organisations: *“Very clear now*, *but that message was garbled along the way*…*we have different guidance from… Public Health England and the internal guidance… So*, *changed*, *continually*, *and it was … very confusing for staff”* (Doctor, A&E, site 1). Participants reported it was easy to forget to wear PPE when moving between different areas of the hospital and when busy: “… *when you’re walking into a clinical area from a non-clinical*, *it can be easy to forget to put your mask on*” (Doctor, A&E, site 1). However, reminders and prompts (e.g., emails and posters) were perceived as enablers.

Most participants perceived training for the safe donning and doffing of PPE to be helpful, including video demonstrations and the opportunity to practice skills. Overall, most participants reported they had the necessary skills to safely don and doff PPE easily, with one participant commenting: *"I don’t think that I typically struggle with putting the mask on safely… there’s been a lot of guidance*. *You wash your hands*, *you put on the mask*, *you take off the mask*, *dispose of it*, *wash your hands again"* (Manager, site 1). However, some participants lacked confidence and/or reported not receive training on PPE with which they had limited prior experience.

*Social opportunity*. Survey responses revealed that participants felt supported by their managers and peers to wear PPE. There was a significant, negative association between the use of PPE and agreement with the statement ‘*My colleagues are not using PPE*’ (M = 1.78, SD = 0.91; R_s_ = -0. 266, *p* = 0.020) (see Table 5).

In interviews, participants reported that wearing PPE negatively impacted interactions with patients: *“…I have a mask on and I have a face shield on and I’m trying to reassure this really scared patient and they can’t see the face behind the mask*. *They can’t see my smile anymore”* (Nurse, ICU, site 1). Two interviewees reported sometimes removing PPE to communicate with patients: *“…in order to sort of facilitate communication*, *there have been times when I’ve… pulled the mask down*.*"* (Doctor, A&E, site 1). Overall, colleagues were generally perceived by participants as having a facilitative role on PPE use. Participants commented that observing their colleagues in PPE often prompted their own behaviour: *“I’d occasionally walk out into the corridor from my office without my mask on*, *but then you’d see a corridor full of masked people and be like*, *oh*, *I’ve forgotten my mask*.*”* (Doctor, Haematology, site 2). Some reported that colleagues also provided practical support by assisting with donning of PPE. A small number of participants commented that they exerted pressure on others when they observed non-adherence: *"it was important for me to set a good example and sometimes to point out where other people were not"* (Nurse, ICU, site 1). In contrast, however, one participant reported that their adherence to PPE guidance was ridiculed by another staff member, resulting in the removal of PPE: *“I have bumped into an infectious diseases consultant I know on two occasions when I’ve been wearing the mask and he’s just laughed at me and I’ve taken it off to have a conversation with him*.*”* (Doctor, site 1).

*Physical opportunity*. Participants indicated in the survey that they had enough time to put on or remove PPE, that PPE was available in convenient locations, and that PPE fit properly (see Table 5). No significant correlations with PPE use were found.

Accordingly, in the interviews, most participants commented there was good availability of PPE, although some specific types of PPE (e.g., visors) were in short supply and the available PPE did not always fit correctly: *“We have a lot of PPE*… *We’ve got a whole storeroom of PPE*. *I don’t think we’ve run out”* (Healthcare Assistant, Acute Medicine, site 2) and “*I’m only small*, *so everything is like quite big on me*” (Doctor, Haematology, site 2). Many participants reported that PPE stations were set up around the hospital making PPE easily accessible: *“We had clear*, *signed areas where to get it”* (Nurse, A&E, site 1). However, a small number of participants reported that PPE was not always accessible which led to the reusing of PPE. Some participants reported there was not enough time to don and doff PPE as recommended: *“…I don’t always follow that if I have to rush somewhere*, *it’s like*, *take off your mask*, *dump it in the bin*, *sanitise and run off to the next thing"* (Manager, site 1). Mandating the use of face masks in non-clinical areas was perceived as an enabler: *"I tried*, *after I came back sick*, *very hard to introduce masking into some areas and it was met with resistance… so it’s good that they’re sort of mandated now*.*"* (Doctor, Infectious Diseases, site 1).

*Reflective motivation*. Survey results indicated that participants felt that PPE was necessary for their role, they were sufficiently at risk or exposed to Covid-19, and using PPE was a priority. No significant correlations with PPE use were identified (see Table 5).

Similarly, in interviews, nearly all participants perceived PPE to reduce the risk of transmission: *“on the one hand*, *it felt as though*, *oh*, *this isn’t enough*, *but then*, *on the other hand*, *it wasn’t like all of the healthcare workers were sort of dropping down with COVID*… *So*, *it must have at least afforded enough protection*.*"* (Doctor, A&E, site 1). However, many participants commented that PPE negatively impacts communication with colleagues: *"… it [facemasks] muffles what you’re trying to say*. *You can’t lipread anybody*. *It makes it harder to hear"* (Nurse, ICU, site 1). PPE also made delivering clinical care difficult, for example, participants described that wearing multiple pairs of gloves hindered the ability to do informal temperature checks and PPE more generally made it more difficult to provide physical and emotional comfort: "*You have three pairs of gloves on*. *How are you going to know how cold they are*, *or how warm they are*? *That’s like the personal… the touches and the little things that you do with your patient*." (Nurse, ICU, site 1).

*Automatic motivation*. No significant associations between automatic motivation and PPE use were found in the survey (see Table 5).

During the interviews, most participants described some degree of physical discomfort associated with wearing PPE, such as pain, feeling too hot, and light-headedness, but this did not usually discourage the use of PPE: *“it doesn’t really bother me*, *apart from it*, *I don’t think it’s very comfortable*, *but it’s just one of those things*. *And*, *I’d rather do it so that… to kind of reduce transmission*.” (Doctor, Haematology, site 2). Occasionally, however, the discomfort did hinder PPE use, with one participant reporting: “*maybe I should be wearing a visor but I just feel like I get too hot and I start to feel like a bit nauseated with it"* (Nurse, ICU, site 1). Wearing PPE for long hours was also reported by some participants to cause feelings of stress and anxiety; and other participants commented that it can leave you thirsty and make it more difficult to take a comfort break. Overall, using PPE had become a habitual behaviour during the pandemic: “*it’s almost got to the point where now you look strange if you don’t have a mask on”* (Doctor, Haematology, site 2).

### Phase 2: Intervention design

The gap analysis and potential BCTs that could increase social distancing by directly targeting one or more COM-B influences are presented in S7 and S8 Tables, respectively. Five intervention strategies were presented at the stakeholder consultation meeting, including: social distancing champions, team competition, communications about consequences of (not) social distancing, digital reporting of room capacity levels, and virtual handovers (see S9 Table). Two intervention strategies (social distancing champions and team competition) were viewed as most implementable, judged to have good practicability, effectiveness, and potential equity. Two strategies (digital reporting of room capacity levels and virtual handovers) were viewed as least implementable, with poorer perceived practicability and effectiveness. One strategy (communication about the consequences of (not) socially distancing) had mixed views on its implementation, with a possible negative consequence (spillover effect) being that communications are leaked outside of the Trust. See S10 Table for a summary of themes mapped onto APEASE criteria.

## Discussion

Our results highlight the complex and multifaceted nature of two key protective behaviours advised throughout the pandemic to decrease hospital transmission of Covid-19: social distancing in non-clinical communal areas and the use of PPE. Where social distancing was perceived by most to be challenging to implement, PPE use was universally high among participating HCWs (working in acute/vulnerable patient areas) in this study. By using theory-based mixed methods and focusing on two specific hospital sites where exposure to patients with COVID-19 was high, we were able to understand the drivers of both these behaviours in a localised context and formulate intervention options for improving social distancing behaviour, the most difficult of these behaviours to implement.

### Understanding influences on behaviour

Social distancing behaviour was significantly associated with psychological capability, physical opportunity, and social opportunity; the importance of motivation also emerged from the qualitative interviews. With respect to psychological capability, HCWs described the challenges associated with there being a lack of clarity about how and where to socially distance and having to keep social distancing in mind all the time, easily forgetting to social distance when around familiar people. This reflects the fact that social distancing was not yet routine practice among some participating HCWs, thus requiring higher attentional demands during an already very stressful period.

Physical space was identified as a key influence on social distancing behaviour in both the quantitative and qualitative data. Interviewees frequently commented that communal areas such as break rooms were too small to accommodate the number of HCWs at a safe distance and equipment such as computers were typically positioned close together, also prohibiting safe distancing. Observations and interviews with HCWs at a US-based hospital reported similar barriers to social distancing in communal areas, and identified various strategies to accommodate distancing, including removing furniture and spacing out computers, limiting the number of people allowed in any given space, and creating additional break rooms [27]. HCWs that we interviewed towards the end of data collection reported similar strategies being implemented in participating hospitals, which made social distancing easier. This highlights the potential benefit of restructuring physical envrionments to facilitate easier adopition of national recommendations, such as social distancing, at a local level by reducing the cognitive burden of remembering to distance on HCWs.

A lack of support or encouragement from managers was found to be associated with less social distancing. There are a number of reasons why social distancing might not have been enforced, including differences in hierarchy (i.e., junior colleagues not challenging the behaviour of senior colleagues), the type of task being carried out (e.g., discussing patient information) and physical space constraints [27]. The lack of enforcement of social distancing behaviour might have strengthened HCWs’ beliefs that this behaviour is less important than other protective behaviours for limiting COVID-19 transmission within hospitals. This was of particular importance during this period of the pandemic: before the availability of vaccination and as the difficulties around social distancing were perceived at the time to be responsible for multiple within hospital outbreaks (personal communication). Therefore, it is important that during future pandemics, leadership use consistent top down messaging to encourage uptake of protevtive behaviours, further enforced by the aforementioned changes to physical environments.

At the beginning of the pandemic when our data were collected, face coverings were rarely worn in non-clinical communal areas and COVID-19 vaccination was not yet available. Therefore, social distancing behaviour should have been prioritised to decrease transmission between healthcare staff. Interview data suggested contradictory beliefs about the perceived efficacy of social distancing. Some believed that poor adherence to social distancing could lead to COVID-19 transmission within the hospital, whereas others indicated that social distancing may not be as effective as other protective behaviours given that not all staff had contracted COVID-19 despite high levels of exposure. This is consistent with other research that found that believing there is ‘no point’ to social distancing when you have frequent contact with COVID-19 patients was associated with close contact with colleagues at work [14]. It has been suggested that a lack of trust in social distancing guidance might have stemmed from frequent guidance changes [27]. This highlights the significance of organisations developing and disseminating consistent, evidence informed guidance to mitigate frequent changes in recommendations where possible, and to ensure clear communication of the rationale for changes where this is unavoidable.

In contrast, PPE use was very high in the participating hospital trusts. However, it should be noted that approximately 12% of participating HCWs did not report using PPE ‘always’ or ‘very frequently’ which is of potentially significant clinical risk. Interviewees identified several factors related to capability, opportunity, and motivation that encouraged PPE use. Most participants felt they knew how to wear PPE appropriately, with many reporting that they received training in how to don and doff PPE safely; although, guidance was inconsistent at times and was updated regularly which has been widely reported by others [28–30]. Previous research has identified that training in general PPE, COVID-19 PPE, or hand hygiene were associated with improved doffing of PPE [31] and overall adherence to PPE [14]. However, observational research has consistently reported errors in donning and doffing PPE during the COVID-19 pandemic [32, 33]. Therefore self-reported capability may not reflect actual capability, which has implications for national policy decisions which should take into account the peripheral support HCWs require on the ground to enact certain protective behaviours.

Participants frequently reflected on the impact of PPE on their interactions with patients, particularly people with a hearing impairment who often rely on lip reading to communicate [34]. Consistent with other research [35, 36], participating HCWs emphasised the negative impact of PPE on non-verbal communication, commenting that it was more difficult to show empathy and compassion towards their patients. To try to reduce the impact of PPE on patient-clinician interactions, researchers have introduced the use of PPE portraits, a small postcard size photo of the HCW that is fastened to their PPE [37, 38]. For future pandemic planning and management, strategies such as this could become standard practice alongside use of PPE to better enhance HCWs’ ability to perform their role.

As well as influencing interactions with patients, participants commented on how the use of face masks, particularly FFP3 masks, made communication with colleagues challenging during the provision of clinical care. Similar difficulties have been reported in surgical theatres [39] and are likely to be experienced in other noisy hospital environments due to the impact of face masks on speech intelligibility [40]. When all staff are in full PPE, participants commented that it can be difficult to know who is in the room and their specific roles. These findings are consistent with other findings that the use of PPE disrupted information flow and resulted in role confusion [41]. In our research, the perceived impact of PPE on communication with patients and colleagues did not deter HCWs from using PPE, but it is an important barrier to overcome if the use of PPE compromises, or is belived to compromise, clinical care.

Most participants reported that they experienced physical discomfort when wearing PPE (e.g., feeling hot, pain, light-headiness), especially when worn for long periods of time. Previous research has reported similar adverse effects following lengthy durations of PPE use [42–44]. In most cases, discomfort did not prohibit the use of PPE in our study, consistent with other research [28]. This might be because there was almost universal agreement from interviewees that PPE was effective in reducing the risk of transmission of COVID-19. It is not known if HCWs could sustain wearing PPE to the extent they have needed to during the pandemic, if the threat of COVID-19 decreased and the perceived benefits did not outweigh the discomfort experienced.

### Intervention design

In line with evidence [45], interventions developed with a theoretical underpinning, such as the one presented for this study, have the potential to be effective in changing behaviours. Consideration of intervention implementation (including context, acceptability, and potential feasibility of delivering the intervention from those delivering and receiving the intervention [46], as done here, can further enhance intervention effectiveness by helping to better translate evidence into practice [46]. The workshops were feasible to carry out in a timely fashion within the context of a rapidly developing pandemic and allowed for local input to enhance implementation of national policy recommendations. By ascertaining the views of stakeholders, we were able to discount potential interventions that seemed appropriate but were viewed as unacceptable, enabling resources to then focus on interventions more readily implementable and likely adopted (in this case ‘social distancing champions’—involving modelling of social distancing and provision of practical support to facilitate this behaviour; and ‘team competition’—involving observing, feeding back, and comparing social distancing across teams, and incentivising the behaviour with reward). BCTs selected to be delivered in these interventions align with other interventions targeting infection control practices. A systematic review [47] identified that comparison of behaviour and feedback and monitoring were the most frequently used groupings of BCTs targeting hand hygiene practices amongst nurses. Similarly, interventions targeting hand hygiene behaviours, that involved modelling of the behaviour and feedback on the behaviour, were found to have a medium, positive effect in a recent meta-analysis [48]. Social distancing interventions have yet to be researched more widely in the literature, and therefore this study presents a first step towards developing novel, context specific interventions, rigorously developed with implementation as a key focus.

### Policy implications

Our behavioural analysis of two protective behaviours during the early phase of the COVID-19 pandemic, prior to the availability of approved vaccine(s), highlights how we can be better prepared to decrease infectious disease transmission in hospital settings and better protect HCWs and their patients during future outbreaks or pandemics. This is of particular importance for health systems and the professionals who work within them, as reduced transmission will ensure safer working and care environments, stronger and better resourced healthcare systems, improved staff morale during similar health pandemics, and a reduction in health, social and economic losses (e.g. see [49]). Specifically, our study helps to highlight how national policies filter down into local contexts and some of the difficulties presented in implementing these, giving scope to make specific, locally relevant recommendations for change that can feed into national as well as local policy.

To encourage the use of social distancing in non-clinical communal areas, we recommend:

- Developing clear and consistent guidance on how to socially distance in non-clinical areas in consultation with HCWs to ensure that it is practical to implement. The guidance should include information on how social distancing should be carried out alongside other protective behaviours like use of PPE.
- Where possible, creating additional communal spaces for HCWs to limit the number of people in an enclosed space at any one time.
- Removing unnecessary furniture from communal areas to enable seating / computers to be spaced apart.
- Creating positive social pressure to enact social distancing through the use of social distancing champions or team competition.

To encourage the use of PPE, we recommend:

- Introducing clear, consistent guidance on how and when to use PPE that is easily accessible to HCWs.
- Ensuring adequate supply of high quality PPE.
- Using prophylactic dressing under respirator masks to improve physical comfort and decrease skin injury when PPE is needed to be worn for long periods of time [50].
- Using PPE portraits to reduce the impact of PPE use on interactions with patients.

Also, simple early warning systems should be implemented that inform HCWs of what PPE and social distancing guidance needs to be followed to improve their capability and opportunity to change behaviour in a rapid manner during future outbreaks.

### Methodological limitations and future directions

The following limitations need to be taken into consideration when interpreting the findings. First, the sample size for the cross-sectional survey study was small, which precluded the use of planned regression analyses to examine the most significant predictors of social distancing in non-clinical communal areas and use of PPE. This response rate likely reflects the fact that our target participants were primarily front-line HCWs who were under extreme work pressures at the time of the survey. The data were from a single timepoint and therefore did not capture changes in response to different threat levels or changes in infection control guidance. Future research involving longitudinal data collection is needed to examine how stable these protective behaviours are, particularly as the pandemic continues to evolve. Common with qualitative research, interviewers will have introduced elements of bias to the data collection and analysis process, in this instance a specific interest to understand drivers of use of personal protective behaviours through a behavioural science lens. As such, two trained and skilled researchers with different disciplinary backgrounds carried out interviews and carried out analysis, alongside a third researcher. This was to try to limit the impact of any bias introduced. Another limitation is that we did not have the opportunity to evaluate the impact of the stakeholder workshops on the implementation of strategies to improve social distancing behaviour as this coincided with the initial COVID-19 vaccine rollout. This exemplifies the challenges of conducting ecologically valid research during a pandemic.

## Conclusions

Building resilience for the future will require in-depth analysis of behaviours and beliefs among the healthcare workforce. Our results can guide future preparedness and responses to future pandemics and outbreaks, as we provide evidence on behaviours and their influencing factors from the critical early phase of the pandemic, prior to vaccine availability and when there were high rates of infection in the community. Our results highlight the value of understanding the drivers of individual protective behaviours separately. PPE use was high irrespective of its negative impact on interactions with patients and colleagues and overall levels of HCW comfort, suggesting that levels of perceived risk combined with social pressure and strong beliefs about the effectiveness of PPE outweighed these negative consequences. In contrast, social distancing in non-clinical communal areas was rarely carried out by participating HCWs, suggesting that greater uncertainty about the effectiveness of this behaviour in the context of less social pressure and physical environmental constraints made it more challenging to implement. Social distancing champions and team competition were viewed as feasible intervention strategies to improve social distancing. Future research is needed to ascertain the effectiveness of such measures on HCW behaviour and subsequently levels of COVID-19 transmission within hospital settings.

## Supporting information

S1 Fig Interview topic guide. (DOCX)

S2 Fig COREQ checklist. (PDF)

S1 Table Overview of behavioural questions in survey. (DOCX)

S2 Table Health care worker perceptions of risk and exposure to COVID-19 at work. (DOCX)

S3 Table Health care worker perceptions of social distancing in communal areas at work. (DOCX)

S4 Table Health care worker perceptions of use of personal protective equipment (PPE) at work. (DOCX)

S5 Table Subthemes related to use of social distancing in communal areas with supporting quotes. (DOCX)

S6 Table Subthemes related to use of personal protective equipment (PPE) with supporting quotes. (DOCX)

S7 Table Behaviour change techniques (BCTs) identified in hospital comms signage. (DOCX)

S8 Table Potential BCTs targeting social distancing in communal areas at one hospital site. (DOCX)

S9 Table Five intervention strategies to promote social distancing in non-clinical communal areas. (DOCX)

S10 Table Stakeholder workshop analysis. (XLSX)

S11 Table STROBE checklist. (DOC)

## Abbreviations

A&E: accident and emergency
BCW: Behaviour Change Wheel
BCT: behaviour change technique
COM-B: Capability, Opportunity, Motivation model of Behaviour
HCW: healthcare worker
ICU: Intensive Care Unit
PPE: personal protective equipment
SAFER: : SARS-CoV-2 Acquisition in Frontline Healthcare Workers—Evaluation to inform Response
