# Supplementary material for: Factors influencing uptake of protective behaviours by healthcare workers in England during the COVID-19 pandemic: A theory-based mixed-methods study
Source: PLoS One. 2024 May 9;19(5):e0299823. doi: 10.1371/journal.pone.0299823 (PMC11081271; doi:10.1371/journal.pone.0299823)
Supplement: S1 Fig — (DOCX) [file pone.0299823.s001.docx]

*S2 Figure.* Interview topic guide

**Section 1: Background and general experience of working during COVID-19**

1. What is your current role?
2. Can you describe for me, what a typical working week looks like for you?

**Section 2: Risk and experience of working during COVID-19 outbreak**

1. Can you start by telling me about your overall experience of working through the COVID-19 pandemic? What has it been like?
2. What have been the biggest challenges of working during the outbreak?
3. Have you had any concerns about coming to work in the hospital during the outbreak?
4. To what extent do you feel COVID-19 poses or posed a risk to you personally?
5. How much is caring for patients with COVID-19 part of your role?
6. Are there any procedures or activities that you do as part of your role that you feel increase your risk of COVID-19?
7. How has the morale and team culture been throughout the COVID-19 outbreak?

**Section 3: Preventive and protective behaviours**

1. Can you tell me about any actions or measures you have taken to try and reduce your risk of COVID-19 at work?
2. Have you been told your antibody status? – To what extent does knowing this result influence how you carry out protective measures like hand hygiene, use of PPE and social distancing at work?
3. To what extent do you feel staff at this hospital are maintaining social distancing when in communal areas?
4. In which communal areas do you think social distancing is not being maintained?
5. Why do you think social distancing is not possible in communal areas? **OR**
6. How much does the layout of communal areas influence whether or not it is possible to socially distance?
7. Have you been given any guidance or recommendations on social distancing in the workplace?
8. How easy or difficult is it to judge if you are 2m/6ft apart from colleagues?
9. How important do you think it is for you and your colleagues to maintain social distancing in communal areas (when it is physically possible)?
10. How does distancing in communal areas impact on your relationship with colleagues?
11. What are the disadvantages or downsides of social distancing in communal areas?
12. Do you ever forget to keep 2m/6ft apart from your colleagues in communal areas?
13. Have you developed any habits or routines to try and maintain social distancing from colleagues in communal areas?
14. How have you found social distancing outside the hospital and has this experience impacted how you socially distance at work?
15. When it is not possible to keep socially distant in communal areas, do you take any additional measures to try and reduce risk of COVID-19?
16. Are there any other factors that we haven’t discussed that you feel influence whether or not you and your colleagues socially distance in communal areas?
17. What could be done to help better support staff to socially distance in communal areas?
18. When/how often do you use PPE in a typical working day?
19. How important is using PPE in your role?
20. How effective do you think using PPE is at reducing risk of COVID-19?
21. In the context of COVID-19, how clear are you on when and how you should use PPE?
22. How easy or difficult is it to put on/remove PPE safely?
23. How does using PPE make you feel?
24. To what extent does using PPE interfere with delivering patient care?
25. Do patients ever influence whether or not you use PPE as indicated? How so?
26. To what extent are PPE supplies readily available in this hospital?
27. Are there ever times when you forget to use PPE at work?
28. Do your colleagues tend to use PPE as indicated? To what extent is wearing PPE the norm among your colleagues?
29. Are there any other factors that we haven’t discussed that you feel influence whether or not you use PPE at work?
30. What could be done to help better support you and your colleagues to use PPE as indicated?

**Part 4: Potential solutions to barriers and enablers**

1. Is your hospital doing anything to support you and your colleagues during the COVID-19 outbreak? What are they doing?
2. Are there any other measures that you think could be done to help reduce the risk and transmission of COVID-19 in this hospital, that are not currently in place?
