# Supplementary material for: Factors influencing uptake of protective behaviours by healthcare workers in England during the COVID-19 pandemic: A theory-based mixed-methods study
Source: PLoS One. 2024 May 9;19(5):e0299823. doi: 10.1371/journal.pone.0299823 (PMC11081271; doi:10.1371/journal.pone.0299823)
Supplement: S1 Table — (DOCX) [file pone.0299823.s003.docx]

*S1 Table.* Overview of behavioural questions in survey

| **Section** | **Statements** | **Response options** |
| --- | --- | --- |
| **Use of personal protective behaviours** | | |
| Social distancing | - In one day, how often do you find yourself in close proximity (i.e. less than 6ft/2m) to other staff members when in communal / non-clinical areas? - When staff are in communal areas and not caring for patients, how often do you think social distancing is being maintained? | - Always, Very Frequently, Sometimes, Occasionally, Rarely, Never |
|  | - In which of the following situations do you find yourself less than 2 metres (6 feet) from other staff members? [tick all that apply] | - Break/rest times, Mealtimes, Meetings, Handovers, After work, Other |
|  | - How difficult is it to keep more than 2 metres (6 feet) apart from your colleagues in the following areas? [multiple areas listed] | - Very difficult, Difficult, Neither easy nor difficult, Easy, Very easy |
| Personal protective equipment (PPE) | - In a typical working day, how often do you use PPE? | - Always, Very Frequently, Occasionally, Rarely, Never |
|  | - What types of PPE do you currently use [tick all that apply]: | - Face masks, FFP 3 Masks, Visors/ Goggles, Gloves, Fluid repellent gowns, Plastic aprons, Other |
|  | - When do you currently use PPE [tick all that apply]: | - During any patient contact (regardless of COVID-19 status) - During contact with any patient suspected of having COVID-19 - During contact with patients confirmed as COVID-19 positive - When walking around the hospital - When working at a desk - When in communal staff areas (e.g. break rooms) - When commuting between home and the hospital - Other |
|  | - How often do you use PPE when you are with colleagues in non-clinical areas? | - Always, Very Frequently, Sometimes, Occasionally, Rarely, Never |
| Other risk reduction measures | - When it is not possible to maintain social distancing in non-clinical areas, do you take additional measures to reduce risk of COVID-19? [tick all that apply] - When it is not possible to use PPE as indicated when you are at work, what other measures do you take to reduce risk of COVID-19? [tick all that apply] | - Hand washing with soap - Hand washing with alcohol-based rub - Using PPE / social distancing - Avoiding touching your face (eyes/ nose/ mouth) - Disinfecting objects and surfaces - Carrying tissues in case you cough or sneeze - Other |
|  | - In your opinion, which is the most important measure to reduce risk of COVID-19 in the hospital? [please tick one] - In your opinion, which risk reduction measure is most difficult for you to do at work? [please tick one] | - Use of PPE in clinical spaces - Use of PPE in non-clinical/communal spaces - Hand washing with soap and water or alcohol-based rub - Social distancing - Disinfecting objects and surfaces - Avoiding touching your face - Other |
| **Factors that influenced use of personal protective behaviours** | | |
| Exposure and risk perception | - Have you had COVID-19? - Has someone else in your team at work had COVID-19? | - Yes (confirmed case of COVID-19) - Yes (suspected case of COVID-19) - No - Unsure - Prefer not to say |
|  | - In a typical working day, how likely are you to come into contact with COVID-19 patients? | - Definitely will, Very likely, Somewhat likely, Probably not, Definitely not |
|  | - How much is caring for COVID-19 patients a part of your role? | - Not at all, A small part, A substantial part, The main part of my role, Unsure or N/A |
|  | - To what extent do you feel the procedures or activities you do as part of your role place you at risk of COVID-19? | - Major risk, Significant risk, Moderate risk, Minor risk, No risk at all, Don’t know |
| Social distancing | - Please think about reasons why you might not stay 2m/6ft apart from your colleagues when in communal staff areas (e.g. break rooms, meeting rooms, corridors, lifts, canteens/cafes, etc.), and rate your agreement with the following statements. [list of COM-B informed statements; see Table 3] | - Strongly disagree, Disagree, Neither agree or disagree, Agree, Strongly agree |
| Personal protective equipment (PPE) | - Please think of reasons why you do not use PPE and rate your agreement with the following statements. [list of COM-B informed statements; see Table 5] | - Strongly disagree, Disagree, Neither agree or disagree, Agree, Strongly agree |
