# Supplementary material for: Factors influencing uptake of protective behaviours by healthcare workers in England during the COVID-19 pandemic: A theory-based mixed-methods study
Source: PLoS One. 2024 May 9;19(5):e0299823. doi: 10.1371/journal.pone.0299823 (PMC11081271; doi:10.1371/journal.pone.0299823)
Supplement: S2 Table — (DOCX) [file pone.0299823.s004.docx]

S3 Table. Health care worker perceptions of risk and exposure to COVID-19 at work

(N = 86)

|  | | N | % | |
| --- | --- | --- | --- | --- |
| EXPOSURE | |  |  | |
| COVID-19 history (personal) | | | |  |
|  | Yes (confirmed) | 14 | 16.3 | |
|  | Yes (suspected) | 5 | 5.8 | |
|  | No | 53 | 61.6 | |
|  | Unsure | 14 | 16.3 | |
| COVID-19 history (team member) | | | | |
|  | Yes (confirmed) | 74 | 86.1 | |
|  | Yes (suspected) | 3 | 3.5 | |
|  | No / unsure | 9 | 10.5 | |
| Likelihood of coming into contact with patients with Covid-19 | | | | |
|  | Definitely will | 23 | 26.7 | |
|  | Very likely | 31 | 36.1 | |
|  | Somewhat likely | 20 | 23.3 | |
|  | Probably not | 11 | 12.8 | |
|  | Definitely not | 1 | 1.2 | |
| How much is caring for COVID-19 patients a part of your role? | | |  | |
|  | Not at all | 9 | 10.5 | |
|  | A small part | 20 | 23.3 | |
|  | A substantial part | 41 | 47.7 | |
|  | The main part of my role | 12 | 14.0 | |
|  | Unsure | 2 | 2.3 | |
|  | N/A | 1 | 1.2 | |
| PERCEIVED RISK | | | |  |
| Perceived risk of Covid19 | |  |  | |
|  | Major risk | 5 | 5.8 | |
|  | Significant risk | 24 | 27.9 | |
|  | Moderate risk | 32 | 37.2 | |
|  | Minor risk | 18 | 20.9 | |
|  | No risk at all | 4 | 4.7 | |
|  | Don’t know | 3 | 3.5 | |
