# Supplementary material for: Factors influencing uptake of protective behaviours by healthcare workers in England during the COVID-19 pandemic: A theory-based mixed-methods study
Source: PLoS One. 2024 May 9;19(5):e0299823. doi: 10.1371/journal.pone.0299823 (PMC11081271; doi:10.1371/journal.pone.0299823)
Supplement: S3 Table — (DOCX) [file pone.0299823.s005.docx]

S4 Table. Health care worker perceptions of social distancing in communal areas at work

(N = 86)

| Frequency of coming into close contact (<2m) with colleagues in communal areas – N (%) | | |
| --- | --- | --- |
|  | Always | 32 (37.2) |
|  | Very Frequently | 41 (47.7) |
|  | Sometimes | 6 (7.0) |
|  | Occasionally | 2 (2.3) |
|  | Rarely | 2 (2.3) |
|  | Never | 3 (3.5) |
| How often do you think social distancing is being maintained in communal areas? – N (%) | |  |
|  | Never | 9 (10.5) |
|  | Rarely | 26 (30.2) |
|  | Occasionally | 15 (17.4) |
|  | Sometimes | 21 (24.4) |
|  | Very Frequently | 12 (14.0) |
|  | Always | 3 (3.5) |
| When is socially distancing difficult? – N (%) | |  |
|  | Break/rest times | 67 (77.9) |
|  | Mealtimes | 58 (67.4) |
|  | Meetings | 51 (59.3) |
|  | Handovers | 69 (80.2) |
|  | After work | 13 (15.1) |
|  | Other | 7 (8.1) |
| *Perceived difficulty social distancing in communal areas – M (SD) | |  |
|  | Meeting rooms | 2.35 (1.11) |
|  | Offices | 2.16 (1.06) |
|  | Corridors | 2.55 (1.13) |
|  | Break rooms | 1.77 (0.92) |
|  | Toilets | 3.58 (1.34) |
|  | Lifts | 2.31 (1.16) |
|  | Changing rooms | 2.36 (1.37) |
|  | Canteens / cafes / restaurants | 3.12 (1.01) |
|  | Outside the building | 3.84 (1.06) |
| Actions taken when not able to socially distance – N (%) | |  |
|  | Hand washing with soap | 71 (82.6) |
|  | Hand washing with alcohol-based rub | 69 (80.2) |
|  | Using PPE | 30 (34.9) |
|  | Avoiding touching face | 68 (79.1) |
|  | Disinfecting objects and surfaces | 56 (65.1) |
|  | Carrying tissues | 29 (33.7) |

** Scale 1-5 (1 = very difficult, 5 = very easy)*
