# Supplementary material for: Factors influencing uptake of protective behaviours by healthcare workers in England during the COVID-19 pandemic: A theory-based mixed-methods study
Source: PLoS One. 2024 May 9;19(5):e0299823. doi: 10.1371/journal.pone.0299823 (PMC11081271; doi:10.1371/journal.pone.0299823)
Supplement: S4 Table — (DOCX) [file pone.0299823.s006.docx]

S5 Table. Health care worker perceptions of use of personal protective equipment (PPE) at work

(N = 86)

|  | | N (%) | |
| --- | --- | --- | --- |
| PPE use | |  |  |
|  | Always | 44 | (51.2) |
|  | Very Frequently | 32 | (37.2) |
|  | Occasionally | 4 | (4.7) |
|  | Rarely | 2 | (2.3) |
|  | Missing data | 4 | (4.7) |
| PPE use in communal areas | |  |  |
|  | Always | 7 | (8.1) |
|  | Very frequently | 9 | (10.5) |
|  | Sometimes | 22 | (25.6) |
|  | Occasionally | 5 | (5.8) |
|  | Rarely | 16 | (18.6) |
|  | Never | 26 | (30.2) |
| Type PPE currently used | |  |  |
|  | Face masks | 76 | (88.4) |
|  | FFP 3 Masks | 41 | (47.7) |
|  | Visors/ Goggles | 45 | (52.3) |
|  | Gloves | 72 | (83.7) |
|  | Fluid repellent gowns | 40 | (46.5) |
|  | Plastic aprons | 73 | (84.9) |
| When PPE currently used | |  |  |
|  | During any patient contact (regardless of Covid-19 status) | 66 | (76.7) |
|  | During contact with any patient suspected of having Covid-19 | 58 | (67.4) |
|  | During contact with patients confirmed as Covid-19 positive | 58 | (67.4) |
|  | When walking around the hospital | 29 | (33.7) |
|  | When working at a desk | 15 | (17.4) |
|  | When in communal staff areas (e.g. break rooms) | 10 | (11.6) |
|  | When commuting between home and the hospital | 18 | (20.9) |
|  | Other | 1 | (1.2) |
| Actions taken when not able to use PPE | |  |  |
|  | Hand washing with soap | 68 | (79.1) |
|  | Hand washing with alcohol-based rub | 68 | (79.1) |
|  | Social distancing | 52 | (60.5) |
|  | Avoiding touching face | 62 | (72.1) |
|  | Disinfecting objects and surfaces | 61 | (70.9) |
|  | Delay patient contact until PPE is available | 43 | (50.0) |
