# Supplementary material for: Factors influencing uptake of protective behaviours by healthcare workers in England during the COVID-19 pandemic: A theory-based mixed-methods study
Source: PLoS One. 2024 May 9;19(5):e0299823. doi: 10.1371/journal.pone.0299823 (PMC11081271; doi:10.1371/journal.pone.0299823)
Supplement: S5 Table — (DOCX) [file pone.0299823.s007.docx]

*S6 Table.* Subthemes related to use of social distancing in communal areas with supporting quotes

| **COM-B Domain and Subtheme** | | **Type of Influence** | **Example quotes** |
| --- | --- | --- | --- |
| **Psychological Capability** | |  |  |
|  | Clarity of guidance on social distancing | Mixed | “…we kept getting like emails, because we’d changed the staff rooms and stuff… now because it’s getting lesser so we would… The matron would send an email saying how many people can be in a room at a given time and accommodating for changing rooms and stuff like that… we do have updates all the time, to know exactly what we can and can’t do.” *(Healthcare Assistant, Acute Medicine, site 2)*  "…this is where the Trust kind of falls down because there doesn’t ever seem to be anything like this or if there is, I never see it, or I can never find it and it never gets emailed." *(Nurse, ICU, site 1)* |
|  | You have to keep social distancing in mind all the time | Barrier | “…it just takes away a little bit of no-one’s really relaxed… you can’t be too relaxed because then you might not be social distancing… you have to keep that in mind all the time it’s a bit, difficult at times” *(Healthcare Assistant, Acute Medicine, site 2)*  "...break rooms, no, people weren’t very conscious… if your friend was sitting on the sofa, would you go and sit on the other end of the sofa? Yeah, I probably would’ve done... Do I sit next to people on a chair? Yes, I do." *(Advanced Clinical Practitioner, Acute Medicine, site 1)* |
|  | Varying ability to judge 2m distance | Mixed | "It’s not difficult. I mean every mature person who has had to work… with rulers or metres… already have a visual idea of… what it means to have two metres of distance between him or her and another person." (*HCW, site 1*)  "…two metres it’s farther than you’d think… I guess it is a little bit difficult" (*Doctor, Acute Medicine, site 1)* |
|  | Increased awareness of social distancing | Enabler | "…we’re reminding ourselves and also people taking the initiative to go, oh actually there’s too many people. It’s like everybody’s much more aware of the situation now than previously." *(Nurse, ICU, site 1)*  "I think I am actually quite aware because sometimes when I’ve felt someone’s too close to me, I’m thinking, well they’re… certainly not two metres from me so it has become sort of an awareness." *(Healthcare Assistant, Infectious Diseases, site 1)* |
|  | Social distancing guidance introduced too late | Barrier | “I think the lifts being limited to like four people only came in about a month ago, and that was basically, once the pandemic was over." *(Doctor, A&E, site 1)*  "…in terms of like social distancing at work, I don’t think that we did that very well, ever. I think the whole distancing thing didn’t really become a thing for a long while." *(Doctor, A&E, site 1)* |
|  | Information overload | Barrier | “I have received a lot of mail, but I haven’t really read, it was like… I’m plenty of… one thousand stuff to do… And a mail about social distancing, so what I have to do, more than two metres, maybe it’s my fault in this case… the information was, all there, but when you’re working 13 hours and you’re thinking about your patient, your family… the email from [SITE 1] is not the first priority” *(Doctor, ICU, site 1)* |
| **Social Opportunity** | |  |  |
|  | Peer pressure | Mixed | "I do think they are taking it seriously, but I don’t feel like it’s actually being enforced by anybody... They mention that we should be doing it, but they just… seem to turn a blind eye when it’s actually not happening." (*Nurse, A&E, site 1)*  “…we all know that it’s all for our health good, it’s all for our safety good and therefore there is no way that a colleague can negatively influence one to what you want to do. We observe the rules of distancing." *(HCW, site 1)* |
|  | Peer acceptance of social distancing | Enabler | “…it depended on maybe how much other people… felt like they were at risk as well.” *(Doctor, Haematology, site 2)* |
|  | Less likely to distance from colleagues they are more familiar with | Barrier | "…there’s something about people that you are particularly attached to, colleagues that you’re particularly attached to, I suspect there’s a bit of a tendency to be less scrupulous." *(Nurse, ICU, site 1)* |
|  | Personal cultural norms are discordant with social distancing | Barrier | "…you have a lot of like groups of people at work. So, for example, like you have a lot of… nurses and… one of them will cook… and they bring it in and they share it." *(Doctor, site 1)* |
|  | Social distancing impacts workplace culture | Barrier | “Isolating completely, eating your lunch by your desk, isolated all the time, it’s not good, it’s not conducive to a healthy, happy workplace.” *(Advanced Nurse Practitioner, Haematology, site 2)* |
|  | Managers support social distancing | Enabler | "…the managers have been really supportive and really good during this time." *(Nurse, Infectious Diseases, site 1)* |
|  | Role modelling | Mixed | “…it’s changing mindsets and the more you do it the more modelling there is of that kind of behaviour from senior people" *(Manager, A&E, site 1)*  "…maybe the fact that my colleagues weren’t socially distancing either had an effect on me." *(Doctor, Acute Medicine, site 1)* |
|  | Feedback from peers | Enabler | "… some people are really, really… “oh you haven’t provided this, and this is not there, and we can’t socially distance”. Which is good because it keeps us on our toes and makes us think about how we can provide those opportunities in changing rooms for meetings." *(Manager, A&E, site 1)* |
| **Physical Opportunity** | |  |  |
|  | Size of physical space | Barrier | “…it’s been almost impossible, it’s been very tough… we have so many people on shift every day, and… because of our footprint in the middle of Central [CITY]… the communal areas, they weren’t big enough for people… and to do a handover for 20 or 30 people, how can you socially distance that, it’s like it’s, impossible.” *(Doctor, A&E, site 1)*  “…there are definitely areas where people work where there just isn’t enough space to be that far apart…it would very frequently be the case where there were more of us in there... to the point where you weren’t able to distance.” *(Doctor, Haematology, site 2)* |
|  | Layout of furniture and office equipment | Mixed | "…it’s about 10 x 5 metre room with maybe eight couches on either wall… four on one wall, four on the other. Then there’s maybe two walls, two tables, maybe 1 x 1 metre… there’s not really any way more than four people could eat at those being socially distanced." *(Doctor, Acute Medicine, site 1)*  "…a lot of the communal spaces now are very clearly set out… with seats taken away or have barriers in place so in those areas… it’s super easy… that’s places like the main lobby and outpatient areas." *(Doctor, Infectious Diseases, site 1)* |
|  | Limiting staff numbers | Enabler | “…the canteen had like a line system… I went in there once and they was like only letting in three people at the time or something… it was maintained as much as possible everywhere.” *(Healthcare Assistant, Acute Medicine, site 2)*  "We did a whole piece on staggered breaktimes because we realised that we had… 21 break spaces at any given time and it was not just the ED nurses, but doctors, radiographers, pharmacists, porters, cleaners, security staff, all of that to consider." *(Manager, A&E, site 1)* |
|  | Access to larger physical spaces | Enabler | “…we’ve got two sort of coffee rooms now. We’ve got more rooms because… the relatives are not staying or they’re not coming… so we’ve taken basically all of the relatives rooms and it’s a coffee room now… we can still do things.” *(Clinical Practice Facilitator, site 1)* |
|  | Social distancing guidance was impractical | Barrier | "People need to get to work in huge numbers, but that little area where the main bank of lifts are, it’s pretty difficult not to have that crowded. And there were signs about… after a while, only four people in a lift or something like that. Well, what are people supposed to do?" *(Nurse, ICU, site 1)*  "Before we couldn’t because… we’re a large group of people." *(Nurse, ICU, site 1)* |
|  | Inconsistent implementation of distancing rules | Barrier | "Within our department, they’ve introduced… staggered break times… only within the last month, but it’s meant to mean that there’s only seven people in the staff room at any one time. How strictly that’s adhered to, I think the management are quite strict about it, but then at handover times, everyone’s in the staff room, so it’s not really that strict… the break times are strict, but… when we’re starting shifts, everyone’s in the staff room." *(Nurse, A&E, site 1)*  "It wasn’t really implemented, certainly within our staffroom. We have over 100 staff… Within the department, one staffroom, so there could be up to like 15 people within the staffroom at any time, just sat around tables together, sat on sofas together… there wasn’t really any measurements at all… within the department as well… we’d be sat at desks together, we’d be sat in sort of like the hub, where we would do handovers, so would be just in there, in corridors… there wasn’t really anything to… not really like one-way systems for us to go walking around, or avoiding each other… I don’t think there was much at all, really. " *(Nurse, A&E, site 1)* |
|  | Floor markings prompt social distancing | Enabler | “…there are markers on the floor to show the spaces that are expected in-between two people that are standing." *(HCW, site 1)*  "…clinics now have squares, so where like squared tape on the floor for one chair to be sat on." *(Advanced Clinical Practitioner, Acute Medicine, site 1)*  "…they’ve made it to socially distanced with like crosses on the floor" (*Doctor, Acute Medicine, site 1)* |
|  | Virtual working | Enabler | "…for like meetings and things like that, they will encourage people to go into like separate rooms to use like Microsoft Office… or things like that… even if like everyone’s still at work." *(Nurse, A&E, site 1)*  “…a Zoom ward round, where we’ll talk through patients, and he’ll be off site. But it’s never quite the same, and often there’s sort of technological issues." *(Doctor, A&E, site 1)*  "…it was anticipated that they’d display on the screen and that’s how the presentations would be done. So, people could sit socially distanced. But due to a technical issue we weren’t able to display on the big screen, so they used a computer screen to do the presentation and we were all sitting there in masks... we’re not set up for these sorts of things." *(Manager, A&E, site 1)* |
|  | Lack of time | Barrier | "…at break time and obviously when we’re getting changed… there’s not a lot of time for us to do that, so if we were waiting for like people to leave that room… it just wouldn’t be possible" *(Nurse, Infectious Diseases, site 1)*  "…the lift… there’s signs to say that there’s four up but sometimes it’ll open and more people are coming out and people get impatient or people need to start their shift, there’s a huge queue for a lift sometimes." *(Healthcare Assistant, Infectious Diseases, site 1)* |
| **Reflective Motivation** | |  |  |
|  | Impact of social distancing on interactions and relationships with colleagues | Mixed | ”You shouldn’t be scared of each other, and should still work in the team, and… be able to have lunch together, and… have a cup of tea together, and… Catch up together, I still think that that is really important… one metre’s, you can still do that, you can still have lunch together one metre apart. That’s okay… as long as you’re mindful of it and… as best as you can, kind of keep to that sensible… I don’t think you should be like, “Oh my God, you’re less than one metre”.” *(Nurse, Haematology, site 2)*  “Saying that, keep socially distanced two metres… as humans… you often are closer and… It’s like a fluid, natural kind of where you stand, and where you are, people saying, “Oh, stand back,” I think it would’ve just put people off... and I think it would affect that natural relationship that you bond as a team.” *(Nurse, Haematology, site 2)*  “I try to do social distancing, but it is hard… if you have… in a room when you’ve got three or four computers and you sort of have your colleagues there to… it’s just like in any office, you feel like… it’s good to have somebody next to you, you can just distract yourself and that kind of stuff. Though when they’ve taken one of the computers out it felt really like… you’ve taken one person out of the office and all that.” *(Clinical Practice Facilitator, site 1)*  "…in like social settings in terms of like the… communal eating area and stuff, if you want to have a private conversation, [laughs] you can’t really do that two metres apart." *(Nurse, A&E, site 1)*  “…sometimes just think… missing that face-to-face interaction… you’re losing out a bit… I always prefer that if there’s something complicated that I need to discuss, either with the lab or one of the consultants, that I’d much rather do it face-to-face than over the phone, just ‘cause I feel like you can maybe get your point across maybe better in person.” *(Doctor, Haematology, site 2)* |
|  | Belief that poor adherence to social distancing can lead to COVID-19 outbreaks | Enabler | "…there’s rumours that there’s a COVID outbreak in the ITU staff because they’re not doing it in the staff room so we’re like, we are not going to end up like them, we are going to do it properly." *(Advanced Clinical Practitioner, A&E, site 1)*  "It didn’t and then because we’ve had a few ICU staff downstairs and I’m like… have they got COVID? They are so on it with their PPE and I was like I bet they’re not in the staff room. We need to be more vigilant, so I think… if there is an outbreak, we need to be really careful." *(Advanced Clinical Practitioner, A&E, site 1)* |
|  | Social distancing less effective than other protective behaviours | Barrier | “I do really wonder about the difference between one metres and two metres, and certainly, if you’ve got a mask on... I think, probably, it doesn’t make a huge difference. I have no evidence for that, but… how come 70-plus per cent of us have been exposed, more than likely, haven’t received it? Well, we must be doing something else that’s important and right.” *(Doctor, Surgery, site 2)*  “At hospital, with each other, we were trying... to be as much polite as we can, but it was like, ‘oh, come on, we already have it, so, doesn’t matter.’” *(Doctor, ICU, site 1)* |
|  | My colleagues aren’t a risk to me | Barrier | "Outside of work you’re conscious of it because everybody else is, but as soon as you come in you’re so used to… I remember one of the doctors say, ‘oh we’re one bubble now, we’re one social bubble.’" *(Manager, A&E, site 1)* |
|  | Contradictory to socially distance in communal areas after not distancing in clinical areas | Barrier | “I think you’re aware that most of the time it’s not possible to be two metres away from each other and then when very occasionally it is possible it seems a bit ridiculous to suddenly take a step backwards.” *(Doctor, site 1)*  "…there’s like a group of 30 nurses, all in the same room... For the handover, without wearing masks, without social distancing, and then, we leave the staffroom, and then we’re told we need to implement it... we have special break times and whatever, but it’s just very contradictory to start the day off in that way." *(Nurse, A&E, site 1)* |
|  | Too late for social distancing to make a difference | Barrier | “…as I said before, in the beginning yes, we should’ve been better at it, but now… I think it’s probably too late.” *(Doctor, A&E, site 1)* |
|  | Social distancing is not a priority over job responsibilities | Barrier | “In my experience people might be keeping several metres apart and wearing masks but then they can’t hear each other, or something gets missed and then they take off the mask and move closer together… you prioritise the job and not the social distancing.” *(Doctor, site 1)*  "When you’re having to have confidential discussions about a patient who’s in close proximity, or to a patient… they have to kind of be in close proximity." *(Nurse, A&E, site 1)* |
|  | Impact on day-to-day workplace activities | Barrier | "…the question is how much that will impact on… productivity and being able to just complete all your other tasks. I think if everyone’s wearing a mask, because the fact that masks are so effective… I would say maybe mask wearing is a priority and then social distancing comes next." *(Doctor, Acute Medicine, site 1)*  "Particularly the meetings like handovers and things like that, it does make it a little bit difficult… In terms of eating lunch, I think that might be a little bit more difficult. Just because, often lunch requires a table and if only two people can eat at a table socially distanced then… unless everybody takes their lunch breaks staggered or something, it’s hard for everyone to eat lunch together.” *(Doctor, Acute Medicine, site 1)* |
| **Automatic Motivation** | |  |  |
|  | Forming habits around social distancing | Mixed | “…at first it feels a bit awkward and strange, but… it quickly becomes a habit… some of the people that I’m closer with… it’s definitely harder." *(Nurse, Infectious Diseases, site 1)*  "I think people do kind of slip back into old habits… I sometimes don’t think it’s deliberate… just, you know forget and… It is hard because like we’ve never… this was just so weird." *(Nurse, ICU, site 1)*  “…at the end of the day, we were just carrying about our, three or four like, just like, “Okay, let’s go to grab a coffee,” and, “What do you think at this, about this patient?” blah blah blah. There is some sugar over there, and you’re just moving your body to take the sugar and you’re going through the other, just because you are used to do that, and you are not thinking about what you are doing physically… you are thinking about your work, or all this stuff around your work, or the research or, or something else. So, it’s just, you are used to stay close the others.” *(Doctor, ICU, site 1)*  “I think we maintain it as much as we can… it’s been a while now so it’s become a part of our routine, we changed the way we work… to try and accommodate it as much as we can, and I think a lot of them did pretty well.” *(Healthcare Assistant, Acute Medicine, Site 2)*  "I’ve got into a routine at the minute, where I do just take my breaks… In this particular room that I know is quiet." *(Nurse, A&E, site 1)* |
|  | Seeking emotional support through human contact | Barrier | “It’s just a bit hard because… we have like formed friendships and stuff and it’s like you want to go closer to speak to somebody and you can’t and it’s just a bit strange or like you can’t give the person a hug. Yeah, it has been strange in that sort of aspect, sort of stay away from me [laughs].” *(Healthcare Assistant, Acute Medicine, site 2)* |
|  | Feeling overwhelmed | Barrier | "…clinical staff often felt a bit overwhelmed by that kind of organisational stuff which is quite kind of basic but they had so many things to think about that keeping on top of the telling staff to space out and keep masks on and all the rest of it, I think sometimes felt like one thing too many." *(Nurse, ICU, site 1)* |
