# Supplementary material for: Factors influencing uptake of protective behaviours by healthcare workers in England during the COVID-19 pandemic: A theory-based mixed-methods study
Source: PLoS One. 2024 May 9;19(5):e0299823. doi: 10.1371/journal.pone.0299823 (PMC11081271; doi:10.1371/journal.pone.0299823)
Supplement: S6 Table — (DOCX) [file pone.0299823.s008.docx]

*S7 Table.* Subthemes related to use of personal protective equipment (PPE) with supporting quotes.

| **COM-B Domain and Subtheme** | | **Type of Influence** | **Example quotes** |
| --- | --- | --- | --- |
| **Psychological Capability** | |  |  |
|  | Clarity and consistency of PPE guidance within and across Trust | Mixed | “Very clear now, but that message was garbled along the way… we have different guidance from… Public Health England and the internal guidance… changed, continually, and it was very confusing for us and very confusing for staff.” *(Doctor, A&E, site 1)*  “I think like for comfort and things they’ve said that when you get into your office or like your space that you would normally share with your colleagues anyway, then you don’t have to wear it.” *(Doctor, Haematology, site 2)*  “…the advice was that, if you’re working solely in your office… you don’t need a mask, and if you’re talking to a colleague two metres apart, and we all were at that point… right at the start… then you don’t need a mask.” *(Nurse, Haematology, site 2)* |
|  | Frequently changing PPE guidance | Mixed | “…there was just too much information that changed too often… it was supposed to be helpful to sort of keep people updated and… not keep people in the dark, but I think it almost went the opposite way.” *(Doctor, Haematology, site 2)*  "I’m quite clear on how I’m meant to do it… it’s just, we get a lot of emails about it and there’s an app… So, you... can check the up-to-date [guidance]" *(Nurse, A&E, site 1)* |
|  | Signage informed staff about what PPE to be used | Enabler | “…there was a lot of signage on the entry to ward, and on the entry to every person’s bay, about what you should be wearing for that particular room, or that particular ward. That was probably the most helpful thing… the signage on it, because that would be like clear straightaway, before you even got to the door, what you’re supposed to be wearing.” *(Doctor, Haematology, site 2)* |
|  | Forgetting to wear recommended PPE | Barrier | “Very easy to forget, when you’re sitting in your office and you’re doing something, and the person in your office is two metres away and they’re also doing something, they’ll have their mask on, then you get up to go out to collect something… and you walk down the corridor, no mask. That is very easy, and all of us have done that. Or, you’re on your way out, and you haven’t put a new mask back on to walk out the door.” *(Doctor, Surgery, site 2)*  "Definitely not a full PPE scenario, but there might’ve been the odd occasion where I’ve gone to like do a set of obs on a patient, and I’ve forgotten to put my gloves and apron on, or, if there’s been an emergency situation." *(Nurse, A&E, site 1)* |
|  | Actively seeking evidence-based information on PPE use | Enabler | "it’s just reassurance… I researched it myself and looked up why the change. I spoke to Infection Control, like in the hospital, to try and understand, ‘cause they were frequently on the ward, and that was really helpful as well." *(Nurse, Infectious Diseases, site 1)* |
| **Physical Capability** | |  |  |
|  | Training in PPE use | Mixed | “…the training was helpful… there was a video sent out, but I actually had face-to-face training, which was better. We had a small group session, where we met with… one of the infection-control nurses. She went through the proper like donning and doffing… that was easier to remember, ‘cause when I watched a video, I just felt like you just forget it.” *(HCW, Haematology, site 2)*  “I still don’t think I take it off properly… you take it off the way that we was shown to take it off… I keep washing in alcohol gel and gloves until I’m out of the cubicle and then I wash and alcohol everything else. I probably haven’t quite got the steps of that down properly, but I am doing it safely if that makes sense. I don’t think I’m spreading germs when I come out of the room despite all the drills and stuff." *(Advanced Clinical Practitioner, A&E, site 1)* |
|  | Have the necessary skills to don and doff PPE | Enabler | "I don’t think that I typically struggle with putting the mask on safely… there’s been a lot of guidance. You wash your hands, you put on the mask, you take off the mask, dispose of it, wash your hands again." *(Manager, A&E, site 1)* |
|  | Training less relevant due to guidance changes | Neutral | “…when we did that donning and doffing was that she did it with the full, everything… the proper, the gowns, rather than just the apron… and the visor and then actually we never ended up wearing those things, so I feel like they’d done the training when the guidance was like one thing, and then it changed.” *(Doctor, Haematology, site 2)* |
| **Social Opportunity** | |  |  |
|  | Impact of PPE on interactions with patients | Mixed | "…because I have a mask on and I have a face shield on and I’m trying to reassure this really scared patient and they can’t see the face behind the mask. They can’t see my smile anymore." *(Nurse, ICU, site 1)*  “…in order to sort of facilitate communication, there have been times when I’ve… pulled the mask down." *(Doctor, A&E, site 1)* |
|  | Peer support | Enabler | "…that’s why at the beginning you had people to try and help you get dressed, so if you forgot maybe like a hairnet or something, someone would be, ‘oh’… when we finish, we just… we put our hands out and I would probably ask, am I all done and someone would pick up on… ‘oh you don’t have an apron’, or ‘you forgot that’" *(Nurse, ICU, site 1)*  “…you do have to do it a few times and you always had somebody who would… when we all started we were watching each other, making sure you’ve got your colleagues back… if someone feels like look I’m doing it the first time, would you mind to watch me if I’m doing it right… we would really be really careful in making sure everyone gets to the routine of donning and doffing really.” *(Clinical Practice Facilitator, site 1)* |
|  | Peer pressure | Mixed | “I have bumped into an infectious diseases consultant I know on two occasions when I’ve been wearing the mask and he’s just laughed at me and I’ve taken it off to have a conversation with him.” *(Doctor, site 1)*  "…it was important for me to set a good example and sometimes to point out where other people were not." *(Nurse, ICU, site 1)* |
|  | Role modelling | Enabler | “There’s too many…people that’d be on your case… if you didn’t put it up... I’d occasionally like walk out into the corridor from my office without my mask on, but then you’d see a corridor full of masked people and be like, ‘oh, I’ve forgotten my mask.’” *(Doctor, Haematology, site 2)*  "It’s definitely the culture now, everyone has a mask on. " *(Nurse, A&E, site 1)* |
| **Physical Opportunity** | |  |  |
|  | PPE supplies | Mixed | “We have a lot of PPE… We’ve got a whole storeroom of PPE. I don’t think we’ve run out…” *(Healthcare Assistant, Acute Medicine, site 2)*  “…the kind of PPE that had the most issues were the visors... there never seemed to be enough of those.” *(Doctor, Haematology, site 2)* |
|  | PPE accessibility | Mixed | “…every one of the patient areas before you entered had like a PPE station, so I just always put on PPE there.” *(Doctor, Acute Medicine, site 1)*  “I think it was just a bit unclear at first where all the masks were, because we weren’t told we had to use them at that point... they’d just told us to use them that afternoon, midway through the shift, it was like, well, where are they?" *(Nurse, A&E, site 1)* |
|  | Fit of PPE | Mixed | “We were all fit-tested, there was no problem with that. We always had enough gowns and gloves in theatre.” *(Doctor, Surgery, site 2)*  “…everything’s like… one size… things are like a one size fits all, apart from the gloves. I’m only small, so everything is like quite big on me… but… I didn’t see that as much of an issue itself.” *(Doctor, Haematology, site 2)* |
|  | Mandating use of face masks in non-clinical areas | Enabler | "I tried, after I came back sick, very hard to introduce masking into some areas and it was met with resistance… masks are uncomfortable, no-one wants to wear them so it’s good that they’re sort of mandated now." *(Doctor, Infectious Diseases, site 1)* |
|  | Time to don and doff PPE as recommended | Mixed | “While I don’t always follow that if I have to rush somewhere, it’s like, take off your mask, dump it in the bin, sanitise and run off to the next, the next thing." *(Manager, A&E, site 1)*  "…if a patient was having a little mini emergency, did we always make sure that we had a fresh outer layer on before rushing over to that patient? I don’t think we did." *(Nurse, ICU, site 1)*  “…they move with the same PPE from one bay to another one, without touching the doors… to save time, because they don’t have to undress and get dressed” *(Doctor, ICU, site 1)* |
|  | Quality of PPE | Neutral | “…they move with the same PPE from one bay to another one, without touching the doors… to save time, because they don’t have to undress and get dressed, and to try to save PPE, because the quality of PPE has decreased a lot during time, right now the PPE is… just really bad, but it is protective.” *(Doctor, ICU, site 1)* |
| **Reflective Motivation** | |  |  |
|  | PPE is effective and makes you feel safe | Enabler | "I believe it’s effective because I feel if it wasn’t effective more of us would have actually contracted this virus and also, we would have been able to spread it very easily." *(Staff Nurse, ICU, site 1)*  "…that felt a bit like we were just being exposed to quite a lot of COVID... There was definitely a feeling of… on the one hand, it felt as though, oh, this isn’t enough, but then, on the other hand, it wasn’t like all of the healthcare workers were sort of dropping down with COVID... So, it must have at least afforded enough protection." *(Doctor, A&E, site 1)*  “It probably does make me feel a bit protected… I don’t think it’s a complete protection… I’ve never thought it’s a complete protection. But… it’s there to protect me, and I would look at it like that. Do I feel 100 per cent safe? Oh, ninety-something per cent safe.” *(Doctor, Surgery, site 2)* |
|  | PPE impact on clinical care | Mixed | "I don’t think it really interferes with like giving medications or doing patient care or anything like that." *(Nurse, ICU, site 1)* |
|  | PPE negatively impacts interactions with colleagues | Neutral | "…you have to poke someone to kind of go, err something’s going on but I don’t know what it is and you’re trying to get the other person’s attention in the room." *(Advanced Clinical Practitioner, A&E, site 1)*  "…wearing PPE which is uncomfortable, it muffles what you’re trying to say. You can’t lipread anybody. It makes it harder to hear. I think you don’t realise how much… body language, you would normally use in your interactions with people." *(Nurse, ICU, site 1)* |
|  | Use of PPE is generating excessive environmental waste | Neutral | "…when we first started... you had to wear a mask, apron and gloves every time you went out and it just felt so wasteful… I guess at the back of my mind I’m thinking about the carbon footprint now of wearing all this PPE and how that’s playing out." *(Manager, A&E, site 1)*  "…it’s wasteful… it’s important to wear it, but… it’s so much stuff that just goes in the bin." *(Nurse, Infectious Diseases, site 1)* |
|  | Lack of faith in recommendations for PPE use | Barrier | "…people were not trusting I don’t think, at least initially, about the recommendations that we were giving which led to… a lack of faith in some of the other recommendations that were being given." *(Doctor, Infectious Diseases, site 1)*  “I think… a lot of people just are… if the particles of the virus are so small, and then when they’re airborne, they become so dilute, why are we in a mask now?... Why was it not right at the heart of COVID?” *(Nurse, Haematology, site 2)*  "…they then tell us to wear gloves and aprons all the time, and it’s like… I’ve not been doing that for the last three weeks, what’s the point in doing it now, kind of thing, or like why are we… only told that now?" *(Nurse, A&E, site 1)* |
|  | People feel a false sense of protection | Barrier | "…the receptionists feel very much that because they’re sitting behind a glass barrier, they don’t necessarily then have to wear a mask. But they’re essentially sitting in the most undifferentiated area of the department... I think people feel a false sense maybe, of protection." *(Manager, A&E, site 1)* |
|  | Wearing PPE is unnecessary for non-clinical tasks | Barrier | "I don’t really see any reason why I have to wear a visor when I’m sat at the computer typing for example.” *(Nurse, ICU, site 1)* |
| **Automatic Motivation** | |  |  |
|  | PPE is uncomfortable to wear | Neutral | “…it doesn’t really bother me… I don’t think it’s very comfortable, but it’s just one of those things… I’d rather do it so that… to kind of reduce transmission.” *(Doctor, Haematology, site 5)*  “Maybe I should be wearing a visor but I just feel like I get too hot and I start to feel like a bit nauseated with it." *(Nurse, ICU, site 1)* |
|  | Wearing PPE has become habit | Enabler | “…it’s almost got to the point where now you look strange if you don’t have a mask on… it is just the way things are now. You notice more if someone hasn’t got a mask on, compared to if they have, whereas it obviously used to be the other way around.” *(Doctor, Haematology, site 2)* |
|  | Wearing PPE can make you stressed and anxious | Neutral | “13 hours of mask could be quite stressing for everybody.” *(Doctor, ICU, site 1)*  "I guess it did make me feel anxious… I think a lot people would probably just have taken their own advice, and I think I personally took the advice of, I’m gonna wear whatever I want to wear, to make myself feel comfortable…" *(Nurse, A&E, site 1)* |
|  | Wearing PPE can leave you feeling thirsty or in need of a comfort break | Neutral | “…you’re in the PPE you’ve suddenly… thinking about when you can drink, because you can’t then go off and have a wee… Or you’re just feeling thirsty as hell and you can’t just take that off while you’ve just put it on… you can’t just waste the PPE.” *(Clinical Practice Facilitator, site 1)* |
|  | Bored of wearing PPE | Neutral | "But I think people just get bored of it… even people say at work “I’m so bored of putting on all this PPE now”." *(Nurse, ICU, site 1)* |
