# Supplementary material for: Factors influencing uptake of protective behaviours by healthcare workers in England during the COVID-19 pandemic: A theory-based mixed-methods study
Source: PLoS One. 2024 May 9;19(5):e0299823. doi: 10.1371/journal.pone.0299823 (PMC11081271; doi:10.1371/journal.pone.0299823)
Supplement: S7 Table — (DOCX) [file pone.0299823.s009.docx]

*S8 Table.* Behaviour change techniques (BCTs) identified in Hospital Comms signage

| BCT | Hospital signage (each location used a different sign with different content) | | | | | | | | | Example | Frequency BCT used across materials |
| --- | --- | --- | --- | --- | --- | --- | --- | --- | --- | --- | --- |
|  | Hospital main entrance | Office staff facing entrance | Bathroom stickers | Meeting rooms | Lift floor stickers | Lift posters | Social distancing floor stickers | Reception desks | Seat stickers |  |  |
| 9.1 Credible source | x | x | x | x |  | x |  |  |  | Hospital logo | 5 |
| 4.1 Instruction on how to perform the behaviour | x | x | x | x | x | x | x | x | x | “keep 2M apart” | 9 |
| 5.3 Information about social and environmental consequences | x | x |  |  |  |  |  |  | x | “Thank you for helping us all to stay safe”  “Thank you for stopping the spread of covid 19 at work”  “Stop the spread of COVID-19” | 3 |
| 5.1 information about health consequences? | x | x |  |  |  |  |  |  | x | “Thank you for helping us all to stay safe”  “Thank you for stopping the spread of covid 19 at work”  “Stop the spread of COVID-19” | 3 |
| 7.1 Prompts/cues | x | x | x | x | x | x | x | x | x | Presence of the signage/stickers | 9 |
| 12.5 Adding objects to the environment | x | x | x | x | x | x | x | x | x | Presence of the signage/stickers | 9 |
| 8.2 Behavioural substitution |  |  |  | x |  | x |  |  |  | “Can you old this meeting virtually?”  “Please use the stairs if you can” | 2 |
