# Supplementary material for: Factors influencing uptake of protective behaviours by healthcare workers in England during the COVID-19 pandemic: A theory-based mixed-methods study
Source: PLoS One. 2024 May 9;19(5):e0299823. doi: 10.1371/journal.pone.0299823 (PMC11081271; doi:10.1371/journal.pone.0299823)
Supplement: S8 Table — (DOCX) [file pone.0299823.s010.docx]

*S9 Table.* Potential BCTs targeting social distancing in communal areas at one hospital site

| **BCT/BCT combination*** | **Example (In the context of hospital site)** | **Barrier/enabler (COM-B domain) targeted by BCT** |
| --- | --- | --- |
| Social support (practical) | Develop a hospital-based app that staff can access/use to report when they enter spaces that are overcrowded (to allow others to avoid these areas) or to report empty spaces (to encourage staff to use these spaces) | “Communal areas are overcrowded; We have nowhere else to go with more space for breaks and meetings” (*Opportunity - Physical*) |
| Prompts/cues | Provide signage that suggests alternative spaces/routes of travel around the hospital to avoid accessing small/overcrowded spaces  Add audible noise/bell/counters that sound each time a person enters a room (may prompt awareness of numbers of people in space) | “Communal areas are overcrowded; We have nowhere else to go with more space for breaks and meetings” (*Opportunity-Physical*)  “I am too busy or in a hurry to think about distancing from colleagues” (*Capability – Psychological*) |
| Restructuring the Physical environment | Provide designated communal areas that are larger in space.  Remove furniture to increase space.  Reduce amount of seating in room to encourage distance.  Use zoom for handovers - designate all meeting rooms to clinical staff at times of handovers in order to encourage use of computers/zoom whilst on hospital site. | “There is not enough space in communal areas to maintain a 2m/ft distance; Furniture in communal areas is too close together; Communal areas are overcrowded; We have nowhere else to go with more space for breaks and meetings” (*Opportunity - Physical*) |
| Restructuring the social environment | Assign social distancing champions to remove/move furniture in spaces. These champions could come from each team and also provide encouragement to staff to maintain distancing and to generate strategies to support distanced support for team morale and cultural practices | “Furniture in communal areas is too close together” (*Opportunity – Physical*)  “Keeping physically apart from my colleagues interferes with team morale and culture; There is lack of support or encouragement from peers to maintain a 2m/6ft distance” (*Opportunity – Social*) |
| Avoiding/reducing exposure to cues to the behaviour | Provide staff with advice such as “avoid crowded areas”.  If appropriate could give staff advice to avoid activities that may lead to poorer social distancing (e.g. “Please avoid face-to-face meetings where possible”. | “Communal areas are overcrowded” (*Opportunity – Physical*) |
| Adding objects to the environment | Add ‘zoom stations’ with laptops/iPad in situ to enable virtual meetings to take place on site. | “There is not enough space in communal areas to maintain a 2m/ft distance; We have nowhere else to go with more space for breaks and meetings” (*Opportunity- Physical*) |
| Identification of self as role model | Use messaging with staff (text alerts/posters/meetings) to provide key message: “What you do impacts what others do”, “Set an example to your colleagues - stay socially distant”. | “Others around me are not maintaining a 2m/6ft distance; There is lack of support or encouragement from peers to maintain a 2m/6ft distance” (*Opportunity – Social*) |
| Identity associated with changed behaviour | Create/foster collective identity for change by getting staff to identify as ‘social distancers’ or ‘spread protectors’ via messaging on posters/intranet/text alerts/could wear badges statin this identify etc. | “Others around me are not maintaining a 2m/6ft distance; Keeping physically apart from my colleagues interferes with team morale and culture; There is lack of support or encouragement from peers to maintain a 2m/6ft distance” (*Opportunity – Social)* |
| Social support (unspecified) | Arrange for the provision of a virtual support group to enable a supportive socially distanced space for staff to access to boost morale.  Arrange for digital events to take place to support staff to observe cultural/religious holidays at a distance.  Encourage virtual meetings (e.g. via zoom/MS teams). | “Keeping physically apart from my colleagues interferes with team morale and culture” (*Opportunity – Social*) |
| Social comparison  +  Social reward | Encourage healthy competition amongst departments to achieve distancing in communal areas by sharing staff comparisons of how social distancing is being maintained on different wards for activities such as meetings and handovers. Also provide verbal/written praise to teams that are actively making changes to social distancing. | “Others around me are not maintaining a 2m/6ft distance; There is lack of support or encouragement from peers to maintain a 2m/6ft distance” (*Opportunity – Social*)  Perceiving social distancing to be a priority (*Motivation – Reflective*) |
| Information about others’ approval | Generate staff testimonies (video/audio/written) or their views on social distancing and their feelings about encouraging this behaviour. | “There is lack of support or encouragement from peers to maintain a 2m/6ft distance” (*Opportunity – Social*) |
| Instruction on how to perform the behaviour | Identify multiple spaces that staff can access for recreational activities and inform staff of where these are and when they should be used. | “We have nowhere else to go with more space for breaks and meetings” (*Opportunity – Physical*) |
| Non-specific incentive/reward | Tell team a reward will be delivered if they achieve good social distancing – the activity should be something valued by the team e.g. a socially distanced coffee morning, hospital logo hoody. | “I am not in a habit of keeping distance from colleagues” (*Motivation – Automatic*) |
| Anticipated regret | Ask staff to think about the degree of regret they will feel if they do not socially distance (and staff rate of infection subsequently increase). | “When I am in communal areas, I just want to relax; I enjoy being close to my colleagues; It is awkward to keep apart from my colleagues” (*Motivation – Automatic*)  Perceiving themselves to be at risk of contracting COVID-19; Perceiving that distancing from colleagues will help to reduce the spread of COVID-19; Perceiving social distancing to be a priority (*Motivation – Reflective*) |
| Reduce negative emotions | Provide staff with advice on how they can continue to relax in some communal areas and maintain closeness even at distance. | “When I am in communal areas, I just want to relax; I enjoy being close to my colleagues; It is awkward to keep apart from my colleagues” (*Motivation – Automatic*) |
| Framing/reframing | Suggest to staff that they might want to think of social distancing as supporting and protecting their colleagues rather than viewing it as something that is awkward to do. | “When I am in communal areas, I just want to relax; I enjoy being close to my colleagues; It is awkward to keep apart from my colleagues” (*Motivation – Automatic*) |
| Social comparison  +  information about health consequences / information about social and environmental consequences  +  credible source  +  feedback on (outcomes of) behaviour | If hospital data was available on this – could show link between distancing and reduction of spread and compare across departments/other hospitals/other settings e.g. if distancing is poorer on one ward and rate of spread Is higher vs another ward where distancing is better, and rate of spread is lower.  Could reinforce this message by providing staff with evidence on risk of Covid and importance and impact of social distancing from sources they feel are credible such as PHE, colleagues, ID leads etc. This information could be delivered via comic strips/ short videos demonstrating the importance of distancing in social/communal spaces e.g. could demonstrate the ease of spread in these areas with a video modelling the spread/social benefits of not spreading amongst colleagues. | Perceiving themselves to be at risk of contracting COVID-19; Perceiving that distancing from colleagues will help to reduce the spread of COVID-19; Perceiving social distancing to be a priority (*Motivation – Reflective*) |
| Problem solving  +  Goal setting (Behaviour)  +  Review Behavioural goal  +  Feedback on behaviour  +  Discrepancy between current behaviour and goal | Encourage staff to discuss how to adapt activities in social/communal areas (e.g. team handovers) to encourage distancing. Ask team members to set one of these suggested adaptations as a goal specifying where, when and how they will do this. Review goal with staff at regular intervals and adjust if needed.  Alongside this, observe distancing in communal areas and record the number of ‘breeches’ of guidance – make staff aware that these observations will be taking place and provide weekly or monthly feedback of observation & updates on areas where social distancing is and is not being observed. When providing this feedback, indicate to staff where their distancing behaviour contradicts any goals set by team to improve distancing. | “Others around me are not maintaining a 2m/6ft distance” (*Opportunity – Social*)  “I am not in a habit of keeping distance from colleagues” (*Motivation- Automatic*)  Perceiving social distancing to be a priority (*Motivation – Reflective*) |
| Goal setting (outcome)  +  Review outcome goals | Set team/department/hospital goals for reducing spread of covid-19 by assessing rates of infection amongst staff (attributable outcome to social distancing). Then review rates of Covid-19 spread between staff thought to be attributable to not following social distancing guidance. Consider adjusting goal as appropriate e.g. we want to see the rate of cases in your team reduce by xx% in next month. | Perceiving that distancing from colleagues will help to reduce the spread of COVID-19; Perceiving social distancing to be a priority (*Motivation – Reflective*) |
| Commitment  and/or  Behavioural contract | Across the hospital – request staff affirm their commitment (could be verbally) using phrases including ‘I will” and ‘high priority’ e.g. I will make social distancing in communal areas one of my highest priorities in order to reduce the spread of Covid-19 amongst my peers  And/or  Ask all staff to sign a contract (should be witnessed) agreeing to/committing to improving social distancing in communal areas of the hospital | Perceiving social distancing to be a priority (*Motivation – Reflective*) |
| Information about others’ approval | Provide testimonies from other staff/credible sources either indicating why they see distancing as a priority and approve of the behaviour, or what they perceive the risks are of not distancing and why they disapprove of not distancing. | Perceiving themselves to be at risk of contracting COVID-19; Perceiving that distancing from colleagues will help to reduce the spread of COVID-19; Perceiving social distancing to be a priority (*Motivation – Reflective*) |
| Comparative imagining pf future outcome(s)  +  Salience of consequences | Prompt staff to imagine/think about the likely outcomes if social distancing across the hospital was improved e.g. better staff health, better ability to maintain independence to go out and about on rest days (as do not have to socially isolate etc).  Alongside this activity, use impactful imagery displaying consequences of achieving social distancing (e.g. image of someone carrying out fun daily activities, image of someone successfully saving someone’s life as part of their job role, videos of staff engaging in external activities e.g. fundraising runs) - moves beyond simply providing information about consequences and progressing towards making the positive consequences of achieving social distancing and not spreading Covid very salient and a focus of staff (i.e. making the behaviour salient in a positive way). | Perceiving social distancing to be a priority (*Motivation – Reflective*) |
| Pros and cons | Ask individual staff/teams to generate lists of pros and cons to social distancing in communal areas. | Perceiving themselves to be at risk of contracting COVID-19; Perceiving that distancing from colleagues will help to reduce the spread of COVID-19; Perceiving social distancing to be a priority (*Motivation – Reflective*) |

** Please note, there are additional strategies (BCTs) that the consulted published tool suggests could tap into the selected COM-B domains, however for the purpose of targeting barriers to increased social distancing, we felt that these BCTs would not help to bring about behaviour change and so these are not included in this table.*
