# Supplementary material for: Factors influencing uptake of protective behaviours by healthcare workers in England during the COVID-19 pandemic: A theory-based mixed-methods study
Source: PLoS One. 2024 May 9;19(5):e0299823. doi: 10.1371/journal.pone.0299823 (PMC11081271; doi:10.1371/journal.pone.0299823)
Supplement: S9 Table — (DOCX) [file pone.0299823.s011.docx]

## *S10 Table.* Five intervention strategies to promote social distancing in non-clinical communal areas.

| **Barrier** | **Who** | **What** | **When** | **Where** | **Why** |
| --- | --- | --- | --- | --- | --- |
| **Social Distancing Champions** | | | | | |
| “Keeping physically apart from my colleagues interferes with team morale and culture; There is lack of support or encouragement from peers to maintain a 2m/6ft distance” (*Social opportunity*) | Implementer = a senior member of staff from each team/ward/specialty; Recipients = all staff | Asked to model good social distancing behaviour & provide practical support to colleagues (e.g., liaise with Estates and Facilities if spaces are overcrowded, encourage teams to maintain distancing) | During meetings, breaks, administrative activities | In communal areas around the hospital (e.g., meeting rooms, break rooms, offices) | The trust/members of management will ask colleagues to express interest in role/identify and ask colleagues to take on role |
| **Team Competition** | | | | | |
| “Others around me are not maintaining a 2m/6ft distance” (*Social opportunity*); Perceiving social distancing to be a priority (*Reflective motivation*); “I am not in a habit of keeping distance from colleagues” (*Automatic Motivation*) | Implementer = Management from each ?team/ward/specialty; Recipients = all staff | Encourage healthy competition amongst departments to achieve distancing in communal areas by:  1) Observing social distancing in hospital communal areas & record when not carried out  2) Providing staff with feedback on social distancing & COVID-19 transmission  3) Sharing staff comparisons of social distancing across wards for activities such as handovers  4) Prompt consideration of pros/cons to distancing in communal areas & how to adapt activities to help distancing. Set 1 adaptation as a goal (where, when, & how). Review & adjust if needed.  Managers to set team improvement goals at Quality & Safety Committee.  5) Providing verbal/written praise to teams making changes to social distancing  6) Providing team rewards for achieving good social distancing (e.g. coffee vouchers) | Monthly observations & feedback + rewards; Goal setting as needed | Communal areas (observation); Feedback/goal setting at team & committee meetings | Share staff success via collecting best practice examples, blogs & intranet news stories, email & card congratulations from SDT/DMs/DCDs, feedback via meetings |
| **Communications about Consequences of (Not) Social Distancing** | | | | | |
| “Others around me are not maintaining a 2m/6ft distance; There is lack of support or encouragement from peers to maintain a 2m/6ft distance” (*Social opportunity*); Perceiving themselves to be at risk of contracting COVID-19; Perceiving that distancing from colleagues will help to reduce the spread of COVID-19; Perceiving social distancing to be a priority (*Reflective motivation*); “When I am in communal areas, I just want to relax; I enjoy being close to my colleagues; It is awkward to keep apart from my colleagues” (*Automatic Motivation*) | Implementer = Comms staff; Recipients = all staff | 1. Explain the difference between working in clinical areas and using communal areas and the benefits of distancing e.g. “*set an example to your colleagues – stay socially distant*”, “*Social distancing supports and protects your colleagues*”, “Vision – what it will be like when we improve social distancing at [the hospital]?”  2. Collect staff testimonies (video/audio/written) on views about social distancing and why it is important.  3. Encourage staff to think about the degree of regret they will feel if they do not socially distance – include data to show impact of when this is not done. | New message each week/month | Physical locations around [the hospital], staff intranet, staff briefings | To be circulated via text alerts, intranet stories, newsletters, blogs, podcasts, emails |
| **Digital reporting of room capacity levels** | | | | | |
| “Communal areas are overcrowded; We have nowhere else to go with more space for breaks and meetings” (*Physical Opportunity*) | Implementer = all staff; Recipients = all staff | A QR code (linked to online form/hospital app) will be placed on each communal space door at [the hospital]. Can use this to report when enter a space that is overcrowded or quiet. Will be able to see live updated by using online form/app to help avoid crowded spaces & use quiet spaces | Entering a communal space | In communal areas around the hospital (e.g., meeting rooms, break rooms, offices) | Online form accessed via QR codes/app |
| **Virtual handovers** | | | | | |
| “There is not enough space in communal areas to maintain a 2m/ft distance; Furniture in communal areas is too close together; Communal areas are overcrowded; We have nowhere else to go with more space for breaks and meetings” (*Physical Opportunity*) | Implementer = ?Team lead on the ward; Recipients = clinical staff | Conduct handovers virtually as opposed to in-person | During handovers | Meeting rooms | Microsoft Teams using Laptops/iPads |
